# Lung CD4^+^ resident memory T cells use airway secretory cells to stimulate and regulate onset of allergic airway neutrophilic disease

**DOI:** 10.1016/j.celrep.2025.115294

**Published:** 2025-02-17

**Authors:** Vijay Raaj Ravi, Filiz T. Korkmaz, Carolina Lyon De Ana, Lu Lu, Feng-Zhi Shao, Christine V. Odom, Kimberly A. Barker, Aditya Ramanujan, Emma N. Niszczak, Wesley N. Goltry, Ian M.C. Martin, Catherine T. Ha, Lee J. Quinton, Matthew R. Jones, Alan Fine, Joshua D. Welch, Felicia Chen, Anna C. Belkina, Joseph P. Mizgerd, Anukul T. Shenoy

**Affiliations:** 1 Department of Microbiology and Immunology, University of Michigan Medical School, Ann Arbor, MI, USA; 2 Pulmonary Center, Boston University Chobanian & Avedisian School of Medicine, Boston, MA, USA; 3 Department of Medicine, University of Massachusetts Chan Medical School, Worcester, MA, USA; 4 Department of Virology, Immunology, and Microbiology, Boston University Chobanian & Avedisian School of Medicine, Boston, MA, USA; 5 Department of Computational Medicine and Bioinformatics, University of Michigan, Ann Arbor, MI, USA; 6 Department of Medicine, Boston University Chobanian & Avedisian School of Medicine, Boston, MA, USA; 7 Department of Pathology and Laboratory Medicine, Boston University Chobanian & Avedisian School of Medicine, Boston, MA, USA; 8 Department of Computer Science and Engineering, University of Michigan, Ann Arbor, MI, USA; 9 Flow Cytometry Core Facility, Boston University Chobanian & Avedisian School of Medicine, Boston, MA, USA; 10 Department of Biochemistry and Cell Biology, Boston University Chobanian & Avedisian School of Medicine, Boston, MA, USA; 11 Department of Internal Medicine, Division of Pulmonary and Critical Care Medicine, University of Michigan Medical School, Ann Arbor, MI, USA; 12 Lead contact

## Abstract

Neutrophilic asthma is a vexing disease, but mechanistic and therapeutic advancements will require better models of allergy-induced airway neutrophilia. Here, we find that periodic ovalbumin (OVA) inhalation in sensitized mice elicits rapid allergic airway inflammation and pathophysiology mimicking neutrophilic asthma. OVA-experienced murine lungs harbor diverse clusters of CD4^+^ resident memory T (T_RM_) cells, including unconventional RORγt^negative/low^ T helper 17 (T_H_17) cells. Acute OVA challenge instigates interleukin (IL)-17A secretion from these T_RM_ cells, driving CXCL5 production from Muc5ac^high^ airway secretory cells, leading to destructive airway neutrophilia. The T_RM_ and epithelial cell signals discovered herein are also observed in adult human asthmatic airways. Epithelial antigen presentation regulates this biology by skewing T_RM_ cells toward T_H_2 and T_H_1 fates so that T_H_1-related interferon (IFN)-γ suppresses IL-17A-driven, CXCL5-mediated airway neutrophilia. Concordantly, *in vivo* IFN-γ supplementation improves disease outcomes. Thus, using our model of neutrophilic asthma, we identify lung epithelial-CD4^+^ T_RM_ cell crosstalk as a key rheostat of allergic airway neutrophilia.

## In brief

A barrier to understanding neutrophilic asthma and developing effective therapies is a lack of animal models. Here, Ravi et al. demonstrate that transient and recurrent inhalation exposure of sensitized mice with ovalbumin mimics key features of human neutrophilic asthma and uncover an “epithelium-lymphocyte-neutrophil” communication triad that regulates this disease.

## Graphical Abstract

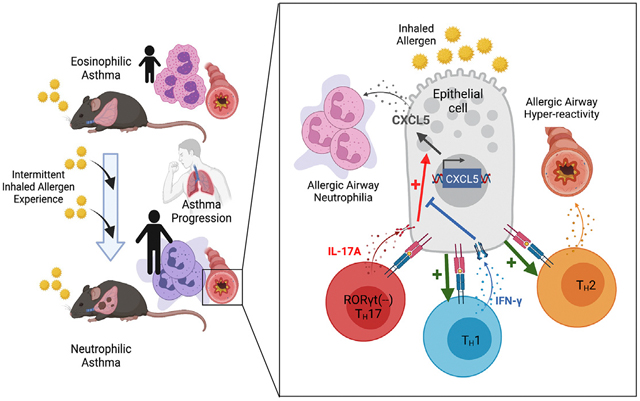

## INTRODUCTION

Asthma is a chronic inflammatory disease of the respiratory tract characterized by goblet cell hyperplasia, mucus hypersecretion, and airway hyper-reactivity, which, if unchecked, can progress to irreversible airway plugging, tissue damage, and death.^1–3^ With ~350 million people affected worldwide, asthma is a major health concern.^4^ Asthma can be eosinophilic or neutrophilic.^5^ While eosinophilic asthma has been extensively modeled in research labs,^6,7^ leading to its recognition as a T helper 2 (T_H_2) cell-driven, steroid-responsive pathophysiology,^1,3^ relatively less is understood about neutrophilic asthma, which often presents as a more severe and steroid-resistant disease.^1,3,5,8^ Furthermore, some patients transition between phenotypes from treatment responsive to treatment resistant for reasons yet unclear.^9–12^ These knowledge gaps underlie fewer therapeutic (Tx) options and poorer quality of life for patients with neutrophilic asthma compared to their eosinophilic counterparts.^3^

Natural and experimental exposures to inhaled allergens seed human and murine lungs with long-lived T_H_2-polarized CD4^+^ resident memory T (T_RM_) cells.^13–19^ These T_H_2 CD4^+^ T_RM_ cells localize around the airways, act as first-line immune sentinels of the experienced lung,^15,19^ and produce more type 2 inflammatory cytokines, like interleukin (IL)-5 and IL-13, compared to circulating counterparts.^19^ Consequently, T_H_2 T_RM_ cells induce hallmark features of eosinophilic asthma, including eosinophil recruitment, mucus production, and airway hyper-reactivity.^15–19^ While these advances have elucidated host factors underlying “T_H_2-high” eosinophilic asthma and informed Txs targeting immune pathways for patients with this disease, key determinants of the more severe neutrophilic endotype of asthma remain uncertain.

A barrier to understanding neutrophilic asthma and the development of effective therapies is a lack of animal models.^3,20^ Traditional models of allergic asthma include sensitizing naive mice to a prototypical allergen followed by pulmonary allergen challenge to induce T_RM_ cells and/or allergic inflammation.^6,7,15–19^ Such models robustly instigate T_H_2-high eosinophilic allergic airway disease but are transient and weak inducers of neutrophilia. Exogenous inflammatory stimuli that elicit neutrophils and/or have T_H_17 adjuvanticity, like cyclic-di-AMP^21^ or complete Freund’s adjuvant,^22–25^ have been added to the allergen exposures to make the allergic inflammation more neutrophilic. However, inciting neutrophils to an allergic response may not replicate allergy-induced neutrophilia, and such neutrophil-inducing adjuvants are unlikely to be triggers of human disease.^3,26^ Alternatively, since our airborne environment harbors endotoxins that may shape lung immunology,^27,28^ models combining lipopolysaccharide (LPS; either exogenously added or existing as contaminant in commercial reagents) with allergen exposures have been used to instigate allergic airway neutrophilia.^29–34^ These models have provided insights into IL-17A,^30^ interferon (IFN)-γ,^21^ and granulocyte colony-stimulating factor (G-CSF)^30^ in allergic airways, but translating the findings from such models to patient settings has had limited success so far.^3,35,36^ Pertinent to this, although adult human lungs are known to be enriched for CD4^+^ T_RM_ cells,^14,37,38^ whether these lymphocytes have causative or mitigating roles in neutrophilic asthma is unclear. Adult humans have different lung CD4^+^ T_RM_ cells than children^39^ and are disproportionately affected by neutrophilic asthma compared to children,^5,8^ with the severity of neutrophilic inflammation predicting worse pulmonary outcomes.^40^ This suggests that types of lung CD4^+^ T_RM_ cells may influence the asthma pathophysiology. CD4^+^ T_RM_ cells can influence lung epithelial cells to enhance neutrophilic inflammation during pneumonia,^41^ but if they do so in settings of allergy is uncertain. Furthermore, whether and how repeated environmental exposure to allergens remodels lung CD4^+^ T_RM_ cells and influences airway allergic disease is unclear.

Because recurrent but transient inhaled exposures to allergens are inherent to progression into adulthood, we hypothesized that multiple intermittent bouts of inhaled allergens would induce the progression of allergic airway disease from eosinophilic to neutrophilic. With that rationale, we establish a mouse model of neutrophilic asthma that resulted from repeated allergen exposures, involving the physiologically relevant route of recurrent exposure (inhalation), inbred animals for which the greatest variety of genetically engineered lines are available (C57BL/6 mice), and a simple model allergen for which a large panoply of antigen-specific immunological tools have been developed (ovalbumin [OVA]). Thus, our approach elicits allergy-induced rapid airway neutrophilia and its resultant pathophysiology within a useful, tractable, and forward-driving animal model. This model allowed us to comprehensively phenotype CD4^+^ T_RM_ cells in the experienced allergic lungs, highlight an unconventional subset of pathogenic T_H_17 T_RM_ cells, and use genetically engineered mice to elucidate an epithelium-lymphocyte-neutrophil communication triad underlying allergic airway neutrophilia. By identifying key cellular and molecular contributors and constrainers of neutrophilic allergic airway disease, we were guided to identify IFN-γ as a potent suppressor of neutrophilic allergic airway disease. Our results now support further investigation of recombinant IFN-γ (rIFN-γ) and/or the pathways this cytokine triggers as approaches to countering neutrophil asthma.

## RESULTS

### Transient and recurrent allergen experience begets rapid allergic airway neutrophilia

**Figure 1.**
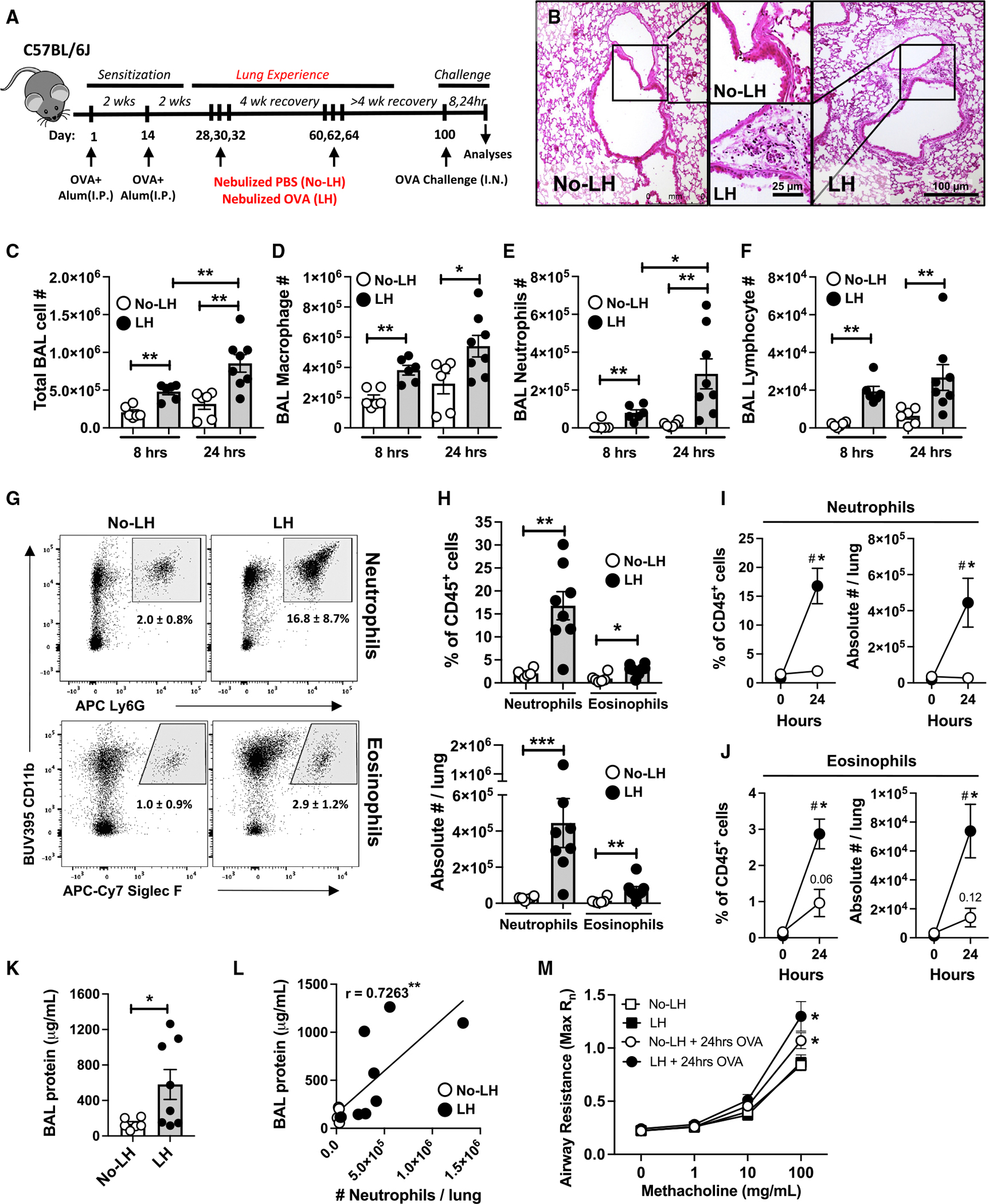
Mouse model of allergic airway neutrophilic disease (A) Schematic of experimental timeline used. (B) Representative images of hematoxylin and eosin-stained sections from mice (*n* = 3 mice) 8 h post-OVA challenge. (C–F) Total cell (C), macrophage (D), neutrophil (E), and lymphocyte (F) numbers in bronchoalveolar lavages (BALs) from mice at designated time points post-OVA challenge. Mann-Whitney test. (G) Representative dot plots depicting lung (ivCD45.2^−^) neutrophils and eosinophils as fraction (%) of CD45^+^ cells 24 h post-OVA challenge; mean ± SD. (H) Numbers of lung (ivCD45.2^−^) neutrophils and eosinophils 24 h post-OVA challenge. Mann-Whitney test. (I and J) Numbers of lung (ivCD45.2^−^) neutrophils (I) and eosinophils (J) in no-LH (white dots) and LH mice (black dots) at baseline and 24 h post-OVA challenge. Two-way ANOVA with Fisher’s least significant difference (LSD) test. (K) Lung damage expressed as BAL protein content. Mann-Whitney test. (L) Scatterplot correlating lung neutrophil numbers and lung damage 24 h post-OVA challenge. Spearman’s correlation coefficient (r) and statistical significance are denoted. (M) Airway reactivity in response to increasing doses of methacholine in mice at baseline and 24 h post-OVA challenge as measured by Flexivent. The measurements represent maximum Rn values. Two-way ANOVA with Fisher’s LSD test. All data have *n* ≥ 5 mice, 2–3 experiments, mean ± SEM, and *p ≤ 0.05, **p ≤ 0.01, and ***p ≤ 0.001.

Relevant environmental exposures make laboratory mice better resemble adult humans in regard to immune cell localizations and activities.^42–47^ Conventional OVA-induced animal models of allergic asthma, relying on sensitized mice being acutely challenged with aerosolized OVA, capture phenotypes of T_H_2 asthma (Figure S1A), including eosinophilic influx with negligible neutrophilia (Figure S1B; gating strategy in Figure S2). Since this model does not account for aeroallergen experiences, which establish and instruct the CD4^+^ T_RM_ cells around human airways,^5,8,14^ we hypothesized that repeated and intermittent exposures of sensitized mice to inhaled allergens over an extended duration will better reflect the human experience^48^ and yield rapid allergic airway disease more characteristic of the neutrophilic asthma that is currently so challenging in humans. C57BL/6J mice were sensitized to OVA in the typical fashion but then administered intermittent recurrent exposures to aerosolized OVA allergen (or PBS vehicle as a negative control) separated by multiple weeks of recovery (to avoid induction of tolerance^49^), before a final challenge with OVA to induce disease exacerbation across all the sensitized mice (Figure 1A). Sets of mice with identical sensitizations and final challenges but differing in inhaled exposures to allergen will hereafter be referred to as lung history (LH) or no lung history (no-LH) groups. Intranasal challenge with OVA induced more severe disease in the LH mice when compared to no-LH mice. LH mice showed severe sickness behavior within 4 h of OVA challenge (Figure S1E). They also exhibited rapid and severe (>5%) loss of body weight within 24 h; this loss persisted through at least 48 h (Figures S1F and S1G). Due to the severity of the disease elicited in LH mice, we hereafter picked the 24 h (or earlier) time points for our challenge studies unless otherwise stated.

Next, we sought to characterize the inflammatory profiles of the mouse airways. Consistent with the rapid onset of severe disease, LH mice displayed more pronounced peribronchial inflammation (Figure 1B) characterized by elevated bronchoalveolar lavage (BAL) cellularity (Figure 1C) that included greater macrophage (Figure 1D), neutrophil (Figure 1E), and lymphocyte (Figure 1F) numbers within 8 h of challenge when compared to no-LH mice. Flow cytometry using intravital staining to exclude circulating leukocytes also revealed greater frequencies and numbers of extravascular (ivCD45.2^−^) lung neutrophils (Figures 1G and 1H), with a modest contribution of eosinophils (Figures 1G and 1H) in the LH mice compared to their no-LH counterparts. Of note, prior to allergen challenge, both LH and no-LH mice had negligible and comparable numbers of extravascular neutrophils and eosinophils in the lung (Figures 1I and 1J), indicating complete resolution of prior inflammation. Furthermore, while allergen exposure increased both neutrophils and eosinophils in the LH mice (Figure 1I), the lungs of no-LH mice displayed modest numerical increases that were confined to eosinophils (Figure 1J).

We considered several limitations. Endotoxin is a human-relevant environmental exposure of interest^27,28^ and independently capable of triggering neutrophil recruitment. Therefore, the robust airway neutrophilia observed in LH mice could result from endotoxin contamination within our OVA preparations.^22^ Such concerns are partially alleviated by the fact that both LH and no-LH mice were challenged with the same OVA preparations but exhibited different outcomes based on their lung history (Figures 1A–1J). Furthermore, naive mice with no prior OVA sensitization showed negligible neutrophilic infiltration after OVA challenge (Figure S1L), suggesting that any possible endotoxin contamination of the OVA preparation was insufficient to induce the robust neutrophilia observed in the LH mice. Another concern is timing. In the standard murine models of OVA-specific allergic airways, a modest and transient neutrophilic infiltration occurs, but eosinophils then become predominant after the allergen challenge.^50,51^ In our study, mice without inhalation histories reflected such cellular dynamics (Figures S1I and S1J). In contrast, our LH mouse model was distinct in that both neutrophils and eosinophils were elevated compared to no-LH controls at all time points (Figures S1I and S1J). Notably, although the airway neutrophilia in the LH mice was rapid and consistently dominant throughout the time course (persisting through at least 48 h post-OVA challenge; Figure S1I), gradual and significant eosinophilic recruitment to the airways was also observed in the LH mice as disease progressed (Figure S1J). Thus, the rapid airway inflammatory response seen in LH mice differs dramatically from that observed in mice without inhalation histories, reflective of immunological remodeling induced by inhaled allergen experience. Indeed, OVA challenge of sensitized mice ushered through the conventional model of eosinophilic asthma (i.e., without lung history) displayed very significantly robust eosinophilic, but weaker neutrophilic, influxes within 24 h when compared to PBS challenge (Figure S1M), an eosinophilic response comparable to that observed in LH mice (Figures 1H and S1M).

Increased vascular permeability and lung edema with plasma leakage into the airways is a feature of asthma.^52^ Consistent with the detrimental nature of neutrophilic inflammation, LH mice demonstrated greater airway edema (measured as BAL protein content; Figure 1K), which positively correlated with airway neutrophilia at 24 h (Figure 1L) and remained high until 48 h (Figure S1K). Of note, both LH and no-LH mice exhibited comparable airway resistance at baseline and presented with exacerbated airway hyper-reactivity to methacholine after inhaled allergen memory recall challenge (Figure 1M). This observation, coupled with the capacity of no-LH mice for rapid eosinophilia (Figure 1J), confirms the utility of sensitized mice without inhaled allergen history (i.e., no-LH mice) as a model of eosinophilic asthma. More importantly, our results demonstrate that transient and recurrent exposure of sensitized mice to inhaled OVA mimics key features of human neutrophilic asthma, yielding a useful allergic animal model for the enhanced neutrophilic inflammation in the airways pertinent to this morbid disease.

### Allergic airway neutrophilia is accompanied by multiple myeloid cell changes

Traditional models of eosinophilic asthma, as shown in Figure S1A, are accompanied by changes to the myeloid landscape involving alveolar macrophages,^53^ monocytes,^54^ CD11b^+^ dendritic cells (DCs),^55^ and eosinophils.^56^ To determine whether myeloid cells were different beyond the neutrophil subset in the LH model evoking neutrophilia, we used flow cytometry analyses of single-cell suspensions from lungs of mice in whom the intravascular leukocytes were discriminated via *in vivo* staining from the intravenous injection of an anti-CD45.2 antibody (Figure S3; gating strategy in Figure S2). At baseline (prior to acute allergen challenge), the LH mice had elevated numbers of tissue-resident interstitial macrophages (Figure S3B), but other lung myeloid cell numbers were comparable to the no-LH mice. Allergen challenge induced elevated recruitment of monocytic cells in LH mice observed as an accumulation of monocyte-derived interstitial macrophages (Figure S3D), Ly6C^+^ inflammatory monocytes (Figure S3E), and Ly6C^−^ patrolling monocytes (Figure S3F) while instigating a modest decrease in the numbers of monocyte-derived alveolar macrophages (Figure S3C). Among the DCs, CD11b^+^ conventional DCs (cDCs) have pathogenic functions in eosinophilic asthma,^57,58^ while plasmacytoid DCs play protective roles.^59–61^ Both of these cell types were expanded in LH mice (Figures S3G and S3H), while no changes in CD103^+^ cDCs (Figure S3I) or CD103^+^CD11b^+^ cDCs (Figure S3J) were observed in the LH lung after allergen challenge. These results together reveal that allergen-experienced lungs have multiple diverse alterations in lung myeloid cells alongside the distinct rapid accumulation of neutrophils.

### Lung cell-derived CXCL5 associates with rapid allergic airway neutrophilia

Considering the neutrophil-attracting chemokines within the lungs of LH and no-LH mice after allergen challenge, both groups showed comparable CXCL1 (Figure S4A) and CXCL2 (Figure S4B), but the LH airways were enriched for CXCL5 (Figure S4C) and diminished for CXCL10 (Figure S4D). CXCL5 correlated with lung neutrophil numbers and BAL protein, but the other CXC chemokines did not (Figures S4E–S4L). To determine the cellular sources of chemokines and how the distinct cells sources were impacted by prior lung allergen history, we measured chemokine transcripts in sorted CD45^+^ leukocytes, CD45^−^EpCAM^+^ epithelial cells, and CD45^−^EpCAM^−^ stromal cells (which include mesenchymal and endothelial cells) from allergen-challenged lungs of LH and no-LH mice. CXCL5 was more strongly expressed in lung epithelial and stromal cells (Figure S4O), consistent with other settings of neutrophilic pulmonary inflammation.^41,62^ Compared to no-LH mice, the LH mice had a higher CXCL5 message in both lung epithelial cells and lung stromal cells but not lung leukocytes (Figure S4O). In contrast, none of the other chemokines were increased in any cell type due to prior inhaled allergen experiences (Figures S4M, S4N, and S4P). Thus, elevated CXCL5 expression by lung epithelial and stromal cells uniquely associates with the rapid airway neutrophilia in allergen-experienced mice.

### Lungs of mice with inhaled allergen history harbor diverse CD4^+^ T_RM_ cells

**Figure 2.**
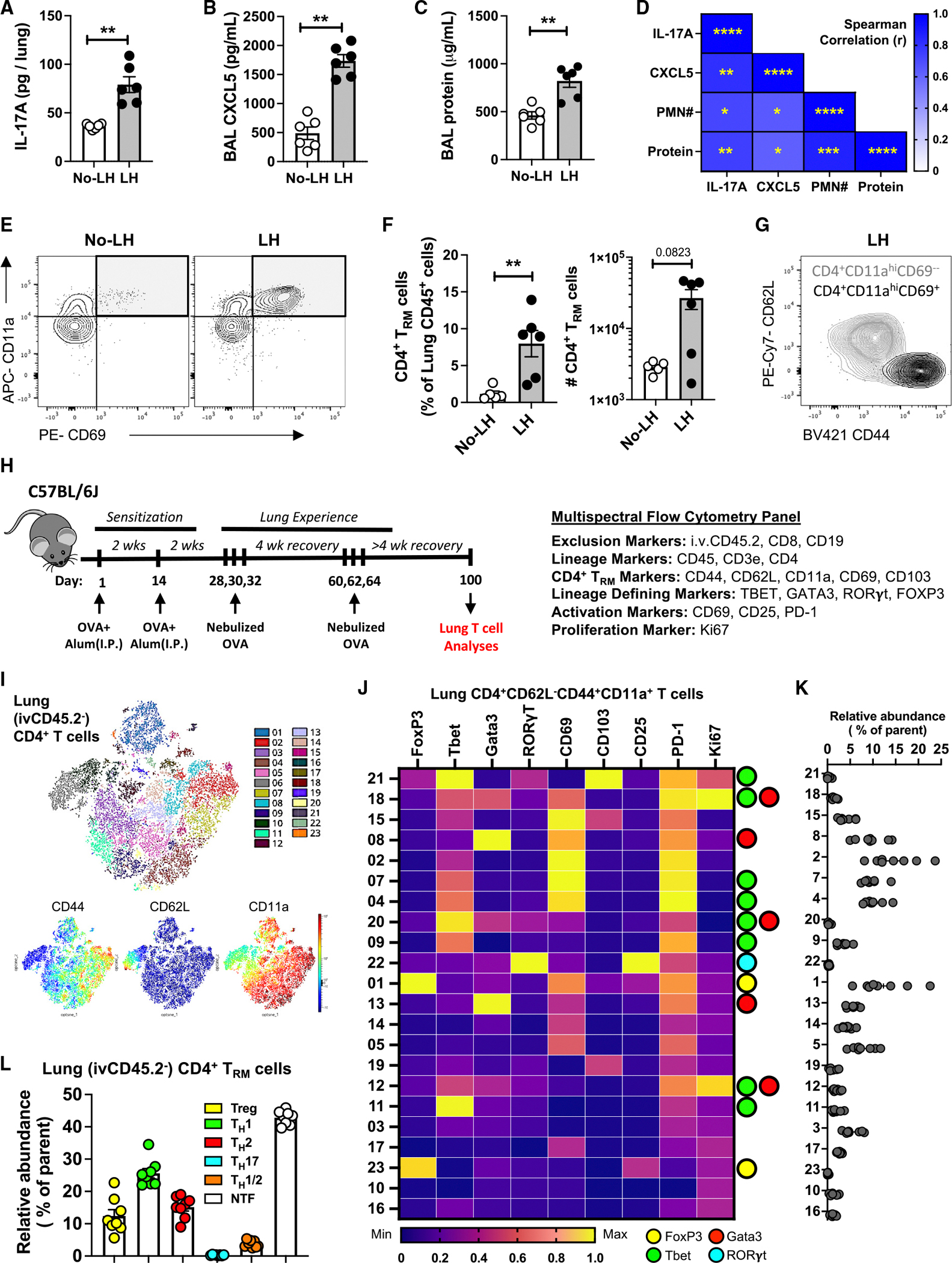
Diverse clusters of tissue-resident CD4^+^ T_RM_ cells reside in lungs of mice with inhaled allergen history (A–C) Levels of whole lung IL-17A (A), BAL CXCL5 (B), and lung damage (C) in mice 8 h post-OVA challenge. Mann-Whitney test. (D) Heatmap depicting Spearman’s correlation coefficients (r) and statistical significance for the specified correlations. (E) Representative contour plots depicting lung (ivCD45.2^−^) CD4^+^ T_RM_ cells identified as CD69^+^CD11a^high^ CD4^+^ T cells in unchallenged mice. (F) Numbers of lung (ivCD45.2^−^) CD4^+^ T_RM_ cells in unchallenged mice. Mann-Whitney test. (G) Representative contour plots illustrating CD62L and CD44 levels on lung (ivCD45.2^−^) CD4^+^ T_RM_ cells (identified as CD69^+^CD11a^high^ CD4^+^ T cells) in comparison to CD69^−^CD11a^high^ CD4^+^ T cells in unchallenged LH mice. (H) Schematic of experimental timeline and antibody panel used. (I) Phenograph clustering overlaid on opt-SNE projection depicting lung (i.v.CD45.2^−^) CD4^+^ T cells concatenated from *n* = 8 LH mouse lungs on day 100. opt-SNE projection with heatmap visualization depicting CD44, CD62L, and CD11a expression levels is shown. (J) Heatmap depicting normalized expression levels of distinct molecules on lung (ivCD45.2^−^) effector memory (CD62L^−^CD44^+^CD11a^+^) CD4^+^ T cell clusters. The lineage-determining transcription factor (LDTF) status of each cluster is depicted on the right. (K) Relative abundance of each cluster in LH mice at day 100 is shown. (L) Frequencies of distinct memory T_H_ cell lineages within lungs of LH mice at day 100. Positivity for a tested LDTF was determined using the cutoffs identified from clusters negative for that LDTF. *p ≤ 0.05, **p ≤ 0.01, ***p ≤ 0.001, and ****p ≤ 0.0001. All data have *n* ≥ 5 mice, 2 experiments, and mean ± SEM.

Respiratory exposure to inhaled antigens leads to the formation of regionally compartmentalized lung-resident CD4^+^ memory T_RM_ cells that, upon subsequent memory recall, secrete lineage-specific cytokines to orchestrate rapid innate immunity.^37^ T_H_17 T_RM_ cell-derived IL-17A augments epithelial CXCL5 production to accelerate neutrophilic inflammation in the lungs during pneumonia.^41,63,64^ Given the rapidity of neutrophil responses (Figure 1E), we examined IL-17A and CXCL5 at the early time point of 8 h after allergen challenge. Both cytokines were already elevated (Figures 2A and 2B), as was BAL protein, characteristic of lung edema (Figure 2C), in the LH compared to no-LH mice. Strong positive correlations were observed among these inflammatory outcomes (Figure 2D). We considered whether T_H_17 T_RM_ cells might also be present in the lungs of mice predisposed to neutrophilic allergic airway disease. Consistent with CD4^+^ T_RM_ cell deposition due to allergen experience, lungs of LH mice were enriched for extravascular CD11a^high^CD69^+^ CD4^+^ T cells (Figures 2E and 2F; gating strategy in Figure S5) that were negative for CD62L and high for CD44 expression (Figure 2G). In contrast, mice ushered though the traditional model of eosinophilic asthma exhibited activated CD4^+^ T cells in their lungs during eosinophilic inflammation but no CD4^+^ T_RM_ cells at baseline (Figures S1C and S1D). Thus, CD4^+^ T_RM_ cells are a salient feature of lungs predisposed to rapid neutrophilic allergic airway disease.

We phenotyped the CD4^+^ T_RM_ cells in experienced lungs to identify helper T cell subsets discriminated by lineage-defining transcription factors (LDTFs), including RORγt, which denotes and mediates T_H_17 cell biology.^65^ We designed a 21-parameter high-dimensional multispectral flow cytometry (MSFC) panel, which probed for LDTFs as well as markers for CD4^+^ T_RM_ cell subsets, activation, and proliferation (Figure 2H). To achieve unbiased identification and quantification of CD4^+^ T_RM_ cell subsets, we computationally concatenated extravascular (ivCD45.2^−^) CD4^+^ T cells from lungs of 8 LH mice, projected the data into two-dimensional space using optimized t-distributed stochastic neighbor embedding (opt-SNE),^66^ and clustered the data in an unsupervised fashion using the Phenograph algorithm.^67,68^ At least 23 distinct clusters of CD4^+^ T cells were observed (numbered in order of decreasing abundance), highlighting an unforeseen complexity within the CD4^+^ T cell pool of lungs susceptible to allergic airway neutrophilia (Figure 2I). Among these, cluster 6 was naive CD4^+^ T cells based on CD62L^+^CD44^−^ staining (Figure 2I) and so was excluded from further analyses to focus on lung memory ivCD45.2^−^CD62L^−^CD44^+^CD11a^+^CD4^+^ cells (Figure 2J). We found two FoxP3^+^ clusters (1 and 23), five Tbet^+^ clusters (4, 7, 9, 11, and 21), two Gata3^+^ clusters (clusters 8 and 13), and three Tbet^+^Gata3^+^ clusters (12,18, and 20) (Figures 2J and 2K), consistent with distinct Treg, T_H_1, T_H_2, and polyfunctional T_H_1/T_H_2 phenotypes, respectively. Only one cluster (cluster 22) was RORγt^+^ and consistent with conventional T_H_17 cells. Many lung CD4^+^ T cells in LH mice lacked all four LDTFs tested (9 of the 22 clusters: 2, 3, 5, 10, 14, 15, 16, 17, and 19) (Figures 2J and 2K). This was surprising since IL-17A led us to anticipate T_H_17 T_RM_ cells. Cumulative frequencies revealed that T_H_1, T_H_2, regulatory T (Treg), and LDTF^−^ T_RM_ cells were common, but T_H_17 T_RM_ cells were vanishingly low (Figure 2L). This contrasts with our studies using the same approach and lungs recovered from pneumococcal pneumonia, in which RORγt^+^ T_H_17 T_RM_ cells are abundant.^69^ As in prior studies,^69^ the blood (ivCD45.2^+^) CD4^+^ T cell subset distributions and surface marker phenotypes differed from the lungs; the blood also lacked RORγt^+^ cells in the LH mice (Figures S6A–S6D). While the no-LH mice failed to accumulate significant lung CD4^+^ T_RM_ cells (Figures 2E and 2F), they possessed circulating CD4^+^ T cells (Figure S6E), CD4^+^ T_CM_ cells (Figure S6F), and CD4^+^ T_EM_ cells (Figure S6G) with similar phenotypes (Figure S6D) compared to LH mice, which reflects the shared experience of systemic sensitization. Thus, although acute disease exacerbation involved robust IL-17A production in the lungs of LH mice, conventional RORγt^+^ T_H_17 T_RM_ cells were not present in these lungs.

### Unconventional T_H_17 T_RM_ cells are sources of IL-17A that drive rapid allergic airway neutrophilia

**Figure 3.**
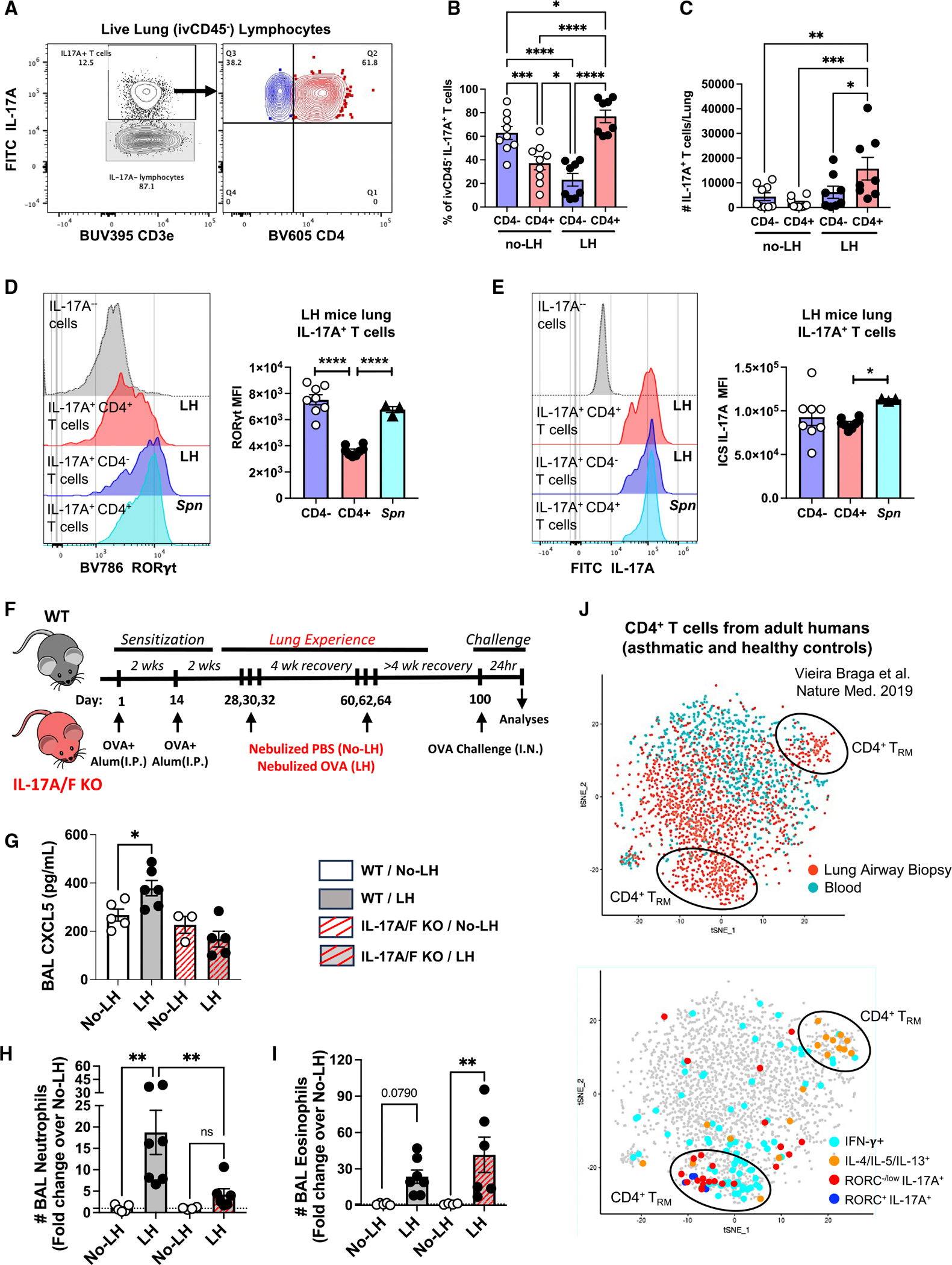
Allergen-experienced lungs include unconventional RORγt^negative/low^ T_H_17 T_RM_ cells that drive rapid allergic airway neutrophilia (A) Representative contour plots depicting IL-17A-producing CD4^−^ and CD4^+^ T cells as identified from live lung (ivCD45.2^−^) lymphocytes within single-cell suspensions of LH mouse lungs stimulated with PMA/ionomycin. (B) Relative frequency of IL-17A^+^ lung (ivCD45.2^−^) CD4^−^ and CD4^+^ T cells in no-LH and LH mice. One-way ANOVA with Fisher’s LSD test. (C) Absolute numbers of IL-17A^+^ lung (ivCD45.2^−^) CD4^−^ and CD4^+^ T cells in no-LH and LH mice. One-way ANOVA with Fisher’s LSD test. (D) Representative histograms and expression levels of RORγt in IL-17A-producing lung (ivCD45.2^−^) CD4^−^ and CD4^+^ T cells in LH mice. One-way ANOVA with Fisher’s LSD test. (E) Representative histograms and expression levels of IL-17A in IL-17A-producing lung (ivCD45.2^−^) CD4^−^ and CD4^+^ T cells in LH mice. One-way ANOVA with Fisher’s LSD test. Expression patterns of *S. pneumoniae*-specific lung (ivCD45.2^−^) CD4^+^ T cells were included as a positive control for conventional T_H_17 signatures. (F) Schematic of experimental timeline used. (G) Bronchoalveolar lavage (BAL) CXCL5 in wild-type (WT) and IL-17A/F-knockout no-LH and LH mice 24 h post-OVA challenge. One-way ANOVA with Fisher’s LSD test. (H and I) Total neutrophil (H) and eosinophil (I) numbers in BALs from WT and IL-17A/F-knockout no-LH and LH mice 24 h post-OVA challenge. One-way ANOVA with Fisher’s LSD test. (J) t-SNE projection of scRNA-seq data depicting expression patterns for T_H_ cell signature markers of interest as expressed by CD4^+^ T cells isolated from airway wall biopsies and peripheral blood of adult asthmatic and healthy humans as displayed on the interactive web portal: https://asthma.cellgeni.sanger.ac.uk/.^14^ *p ≤ 0.05, **p ≤ 0.01, ***p ≤ 0.001, and ****p ≤ 0.0001. All data in (A)–(I) have *n* ≥ 5 mice, 2 experiments, and mean ± SEM.

Because the prompt appearance of IL-17A in lungs without RORγt^+^ CD4^+^ T_RM_ cells was surprising, we sought to determine whether and which lymphocytes from these lungs might be poised to produce IL-17A. We stimulated single-cell suspensions from lungs of LH and no-LH mice *ex vivo*, which induced IL-17A expression from both CD4^−^ and CD4^+^ T cells (Figures 3A and 3B). While CD4^−^ T cells were the predominant source of IL-17A in the no-LH lungs (Figure 3B), most of the IL-17A producers in the LH lungs were CD4^+^ T cells (Figure 3B). This was supported by significant enlargement of the IL-17A-producing CD4^+^ T cell pool in the LH lungs (Figure 3C). Surprisingly, these IL-17A^+^ CD4^+^ T cells from LH lungs expressed less RORγt than the CD4^−^ T cells from the same lungs or the CD4^+^ T cells from lungs of mice recovered from *S. pneumoniae* infections (Figure 3D)^69^ despite comparable IL-17A expression (Figure 3E). Furthermore, these IL-17A-producing CD4^+^ T cells from LH lungs were distinct from the other traditional helper cell subsets in that they did not contain Tbet or Gata3, nor did they co-express IFN-γ, IL-5, or IL-13 (Figures S7A and S7B). While the blood of LH mice also possessed CD4^−^ and RORγt^negative/low^ CD4^+^ T cells that could produce IL-17A (Figures S7C and S7D), their numbers were comparable to blood of no-LH mice (Figure S7E), which responded with poor IL-17A secretion on memory recall challenge *in vivo* (Figure 2A). These findings suggest that lungs (but not blood) of mice with allergen experience are enriched for RORγt^negative/low^ T_H_17 T_RM_ cells that promptly secrete IL-17A to drive CXCL5 secretion and rapid allergic airway neutrophilia upon activation. Indeed, depletion of CD4^+^ cells just before final allergen challenge (Figure S8A) or genetic ablation of IL-17A/F (Figure 3F) compromised CXCL5 accumulation (Figures 3G and S8B) and allergic neutrophilia (Figures 3H, S8C, and S8D), but not eosinophilia (Figure 3I), in the airways of LH mice despite their extensive inhalation experience. Furthermore, consistent with a role of CD4^+^ T cell-driven antigen specificity and not just generalized innate immune memory in this biology, challenging mice with an irrelevant antigen did not exacerbate airway neutrophilia (Figure S9).

Given these findings, we sought to mine independent single-cell RNA sequencing (scRNA-seq) databases from asthmatic human^14,70^ and allergic murine^17^ airways for the presence of the unconventional RORγt^negative/low^ T_H_17 T_RM_ cells. Consistent with our findings in LH mice, interrogation of a scRNA-seq dataset profiling CD4^+^ T cells from airway wall biopsies and peripheral blood samples of adult asthmatic and healthy humans^14^ showed that the airways (and not blood) of adult humans are also enriched for RORγt^negative/low^ T_H_17 T_RM_ cells in addition to T_H_1, T_H_2, and Treg T_RM_ cells (Figures 3J and S10) and neutrophils but not eosinophils.^14^ Reanalyses of a separate scRNA-seq dataset of adult asthmatic and healthy human airways 24 h post-segmental allergen challenge^70^ also confirmed the presence of RORγt^negative/low^ T_H_17 T_RM_ cells in human airways (Figures S11A–S11C). Of note, the unconventional T_H_17 T_RM_ cells that were enriched in our mouse model (~6% of all lung CD4^+^ T cells) and human airways (>2% of all lung CD4^+^ T cells; Figure S11C) were vanishingly scant in murine airways with allergic airway eosinophilia^17^ (<0.8% of airway CD4^+^ T cells; Figures S11D–S11F). Thus, adult human lungs contain T_H_17 T_RM_ cells prone to IL-17A expression despite little to no RORγt, as were identified in mice with a history of inhaled allergen exposures and a predisposition to rapid neutrophilic allergic airway disease.

### Airway Muc5ac^high^ secretory epithelial cells communicate with CD4^+^ T cells and neutrophils

**Figure 4.**
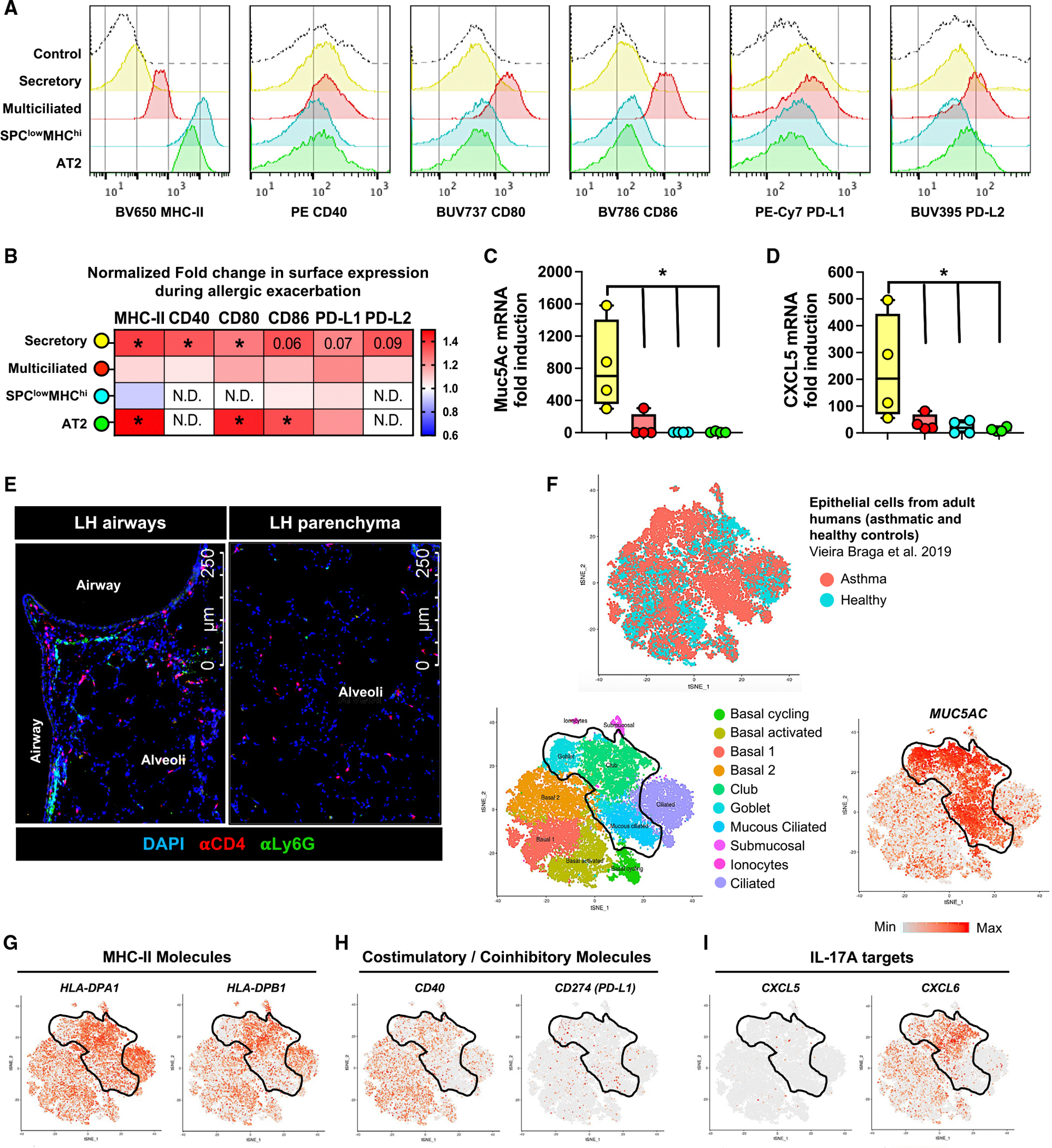
Muc5ac^high^ airway secretory cells communicate with CD4^+^ T cells and neutrophils in inhaled-allergen-experienced lungs (A) Representative histogram plots depicting surface expression patterns of MHC class II, costimulatory molecules CD40, CD80, and CD86, and coinhibitory molecules PD-L1 and PD-L2 on distinct epithelial cells from LH mice at baseline. (B) Heatmap depicting fold change in surface expression levels of specified APC-related molecules on distinct epithelial cells from LH mice 24 h post-OVA challenge normalized to their baseline counterparts. N.D., not detected above FMO control. Mann-Whitney test. (C and D) mRNA levels of Muc5ac (C) and CXCL5 (D) in fluorescence-activated cell-sorted epithelial cells isolated from LH mice 24 h post-OVA challenge. One-way ANOVA with Fisher’s LSD test. *p ≤ 0.05. (E) Representative immunofluorescent micrographs showing anatomical location of Scgb1a1^+^ secretory epithelial cells (green), CD4^+^ T cells (red), and Ly6G^+^ neutrophils (magenta) in LH lungs 8 h post-OVA challenge. DAPI (blue) was used as a counterstain to identify lung structures. Data represent *n* = 3 mice/time point, two experiments. (F–I) t-SNE projection of scRNA-seq data depicting expression patterns for designated markers of interest by different subsets of epithelial cells identified in airways of adult asthmatic and healthy humans as displayed on the interactive web portal: https://asthma.cellgeni.sanger.ac.uk/.^14^ All data in (B)–(D) have *n* = 4 mice, 2 experiments, and mean ± SEM.

Lung epithelial cells use major histocompatibility complex (MHC) class II to function as antigen-presenting cells (APCs) for CD4^+^ T cells during pneumonia.^69^ The roles and dynamics of epithelial MHC class II in allergic lung disease are unknown. We examined professional APC-related molecules on epithelial cells isolated from LH mice at baseline and 24 h after allergen challenge. Mice expressing GFP under the control of human surfactant protein C (SPC) promoter helped distinguish cell types (Figure S12A),^71^ including SPC^low^MHC^high^ alveolar epithelial cells that are the highest MHC class II expressors in resting mouse lungs.^69^ At baseline, alveolar epithelial cells had higher expression of MHC class II, as expected,^69^ while epithelial cells from the conducting airways tended to have higher expression of the other molecules examined (Figures 4A and S12B). Allergen challenge of LH lungs enhanced the expression of all tested molecules in secretory cells from the airways (Figures 4B and S12B), which includes club cells and goblet cells. Type 2 alveolar epithelial cells increased the expression of MHC class II, CD80, and CD86 after allergen challenge, whereas no changes were observed for multiciliated cells or SPC^low^MHC^high^ cells after the allergen challenge (Figures 4B and S12B). The airway secretory cells were the most consistently changed epithelial subset due to allergen challenge (Figure 4B), which is of interest since asthma pathophysiology particularly involves conducting airways and their secretory cells. In the allergen-challenged airways, secretory cells were the sole expressers of Muc5ac (Figure 4C), as expected. In addition, these cells were the only epithelial source of CXCL5 (Figure 4D), the neutrophil-attracting chemokine that was exacerbated by inhaled allergen history (Figures 2B and S4). These findings suggest that Muc5ac^high^ secretory epithelial cells organize immunopathologic niches containing CD4^+^ T_RM_ cells and neutrophils around allergic airways during neutrophilic asthma. Indeed, immunofluorescence analyses of LH lungs 8 h post-allergen challenge revealed biased localization of CD4^+^ cells and neutrophils near the airway, but not the alveolar, epithelium (Figure 4E). Furthermore, exploration of scRNA-seq data profiling airway epithelial cells from adult asthmatic and healthy human lungs confirmed murine findings and revealed that Muc5ac^high^ airway secretory cells from human subjects (Figure 4F) also expressed MHC class II (Figures 4G and S12C), MHC class II-related accessory and costimulatory molecules (Figures 4H, S12D, and S12E), IL-17A receptor components (Figure S12F), and CXCL6, which is the human ortholog of murine CXCL5 (Figure 4I).^14,72^ Muc5ac^high^ secretory cells in adult human airways expressed very little to no inhibitory accessory molecule human leukocyte antigen histocompatibility complex DO (HLA-DO) (Figure S12D), suggesting active antigen presentation in these cells at the time of isolation. Also, CD80, CD86, and PD-L2 expression was minimal, suggesting species-specific differences in airway epithelial biology (Figure S12E). Of note, a pan-epithelial survey of scRNA-seq data from the same study confirmed that Muc5ac^high^ airway secretory cells were high expressors of these immune-facing molecules among all the epithelial cells within adult human lungs (Figure S13),^14^ and a cross-comparison of these signals in a different adult human lung scRNA-seq dataset also confirmed our findings (Figure S14).^73^ Thus, as the only epithelial cells showing elevations across the antigen presentation proteins measured, and the primary epithelial source of the neutrophil chemokines CXCL5/CXCL6, the Muc5ac^high^ airway secretory cells bridge CD4^+^ T_RM_ cells and neutrophils in lungs with extensive inhalation histories.

### Antigen presentation by epithelial cells governs CD4^+^ T_RM_ cell activities

**Figure 5.**
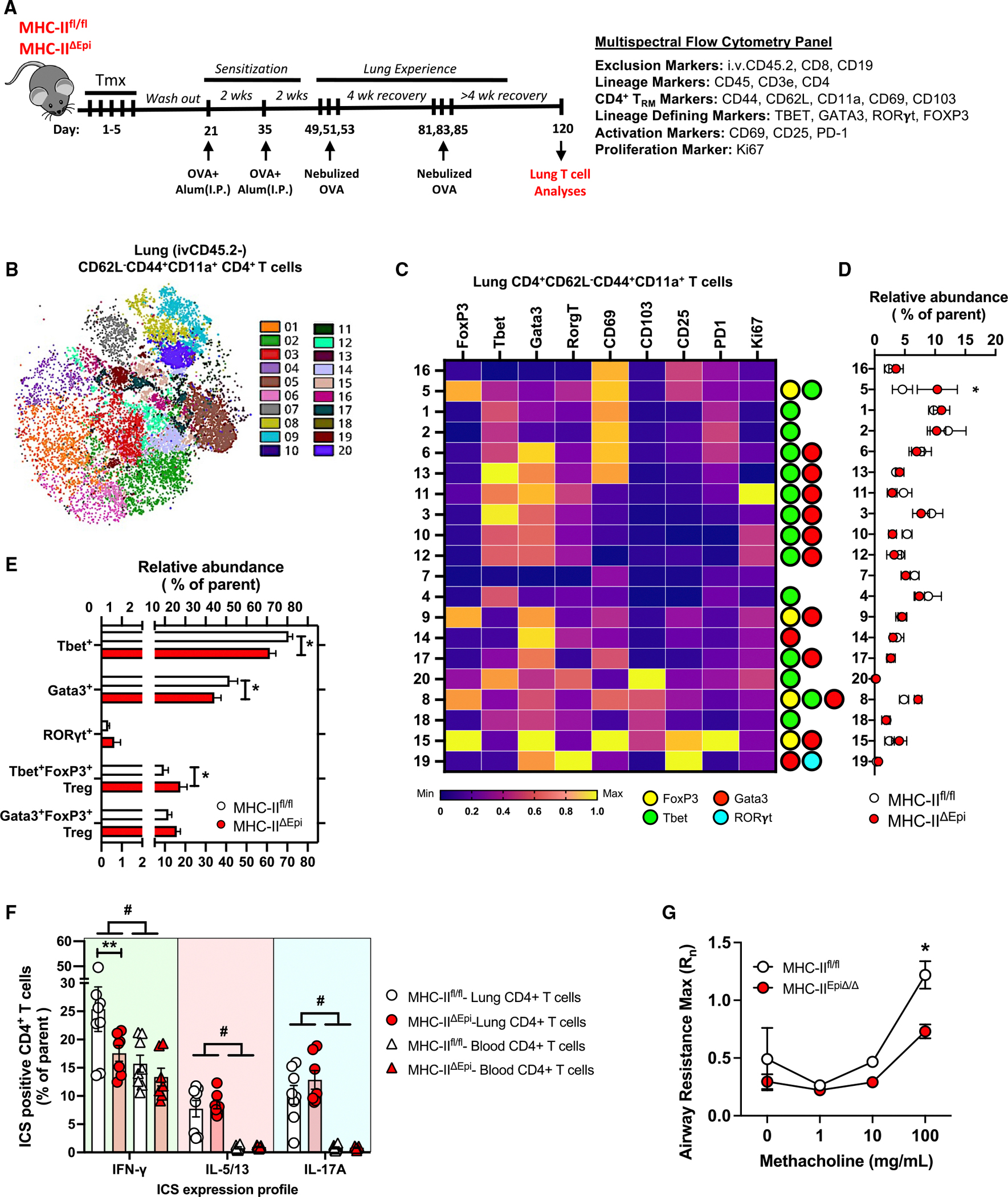
Antigen presentation by epithelial cells governs CD4^+^ T_RM_ cells in allergen-experienced lungs (A) Schematic of experimental timeline and antibody panel used. (B) Phenograph clustering overlaid on opt-SNE projection depicting lung (ivCD45.2^−^) effector memory (CD62L^−^CD44^+^CD11a^+^) CD4^+^ T cells concatenated from MHC class II^fl/fl^ and MHC class II^ΔEpi^ LH lungs on day 120. (C) Heatmap depicting normalized expression levels of distinct molecules on lung effector memory CD4^+^ T cell clusters. Lineage-determining transcription factor (LDTF) status of each cluster is depicted on the right. (D) Relative abundance of each cluster of lung effector memory CD4^+^ T cells in MHC class II^fl/fl^ and MHC class II^ΔEpi^ LH mice at day 120. Two-way ANOVA with two-stage step-up method of Benjamini, Krieger, and Yekutieli to correct for multiple comparisons. False discovery rate (FDR) *q ≤ 0.05. (E) Cumulative frequencies for relative abundances of CD4^+^ T_RM_ cells positive for specified LDTFs. Two-way ANOVA with Fisher’s LSD test. (F) Intracellular cytokine staining (ICS) profile of lung (i.v.CD45.2^−^) and blood (i.v.CD45.2^+^) CD4^+^ T cells isolated from MHC class II^fl/fl^ and MHC class II^ΔEpi^ LH mice on day 120 and stimulated with PMA/ionomycin *ex vivo*. Two-way ANOVA with Fisher’s LSD test. ^#^*p* ≤ 0.05 comparison between lung and blood, *p ≤ 0.05 genotype-dependent comparison within lungs, ^Φ^*p* ≤ 0.05 genotype-dependent comparison within blood. (G) Airway reactivity in response to increasing doses of methacholine in mice at baseline as measured by Flexivent. The measurements represent maximum Rn values. Two-way ANOVA with Fisher’s LSD test. *p ≤ 0.05 and **p ≤ 0.01. All data have *n* ≥ 5 mice, 2–3 experiments, and mean ± SEM.

To test whether epithelial antigen presentation impacts CD4^+^ T_RM_ cells in the lungs with inhaled allergen history, we studied LH mice lacking MHC class II specifically in lung epithelial cells,^69^ referred to as MHC class II^ΔEpi^ mice, after tamoxifen-induced gene targeting. MHC class II^ΔEpi^ mice and similarly tamoxifen-treated but Cre^−^ littermate MHC class II^fl/fl^ mice were ushered through the regimen of sensitization and inhaled allergen exposures, after which lung CD4^+^ T_RM_ cells were phenotyped (Figure 5A). No differences in the abundance of CD4^+^ T_RM_ cells were observed between genotypes (Figure S15A). Phenograph clustering of concatenated lung CD4^+^ T_RM_ cells (ivCD45.2^−^CD62L^−^CD44^+^CD11a^+^) identified 20 distinct clusters (Figures 5B and 5C), none of which were unique to either genotype. Only 1 small RORγt^+^ cluster (cluster 19) was observed (Figures 5C and 5D), which, in these lungs, also expressed Gata3^+^ (T_H_2/17). Deletion of epithelial MHC class II very modestly perturbed CD4^+^ T_RM_ cell abundances on a per-cluster level (Figures 5D and S15B). However, enumeration of CD4^+^ T_RM_ cells sharing common helper T cell phenotypes (based on the expression of individual LDTFs) revealed significant reductions in Tbet^+^ CD4^+^ T_RM_ cells except for those co-expressing Foxp3, which instead increased (Figure 5E). Because Tbet^+^ Foxp3^+^ Treg cells potently suppress Tbet-dependent T_H_1 responses,^74^ our results suggest that the deletion of epithelial MHC class II leads to an expansion of Treg T_RM_ cells to suppress T_H_1 T_RM_ cell activity. Indeed, correlation analyses revealed a strong inverse correlation between these CD4^+^ T cell types (Figure S15C), and *ex vivo* stimulation revealed a 30% reduction in IFN-γ-secreting lung CD4^+^ T cells (Figure 5F). MHC class II^ΔEpi^ lungs also revealed reductions in Gata3^+^ T_RM_ cells (Figure 5E), suggesting dampened T_H_2 T_RM_ responses in LH lungs devoid of epithelial MHC class II. Consistent with T_H_2 cells being potent inducers of asthmatic airway remodeling,^1,3,19^ MHC class II^ΔEpi^ lungs exhibited milder airway hyper-reactivity to methacholine compared to their MHC class II-sufficient counterparts (Figure 5G). Notably, no genotype-dependent differences in the cytokine secretion profiles of blood CD4^+^ T cells (Figure 5F), lung CD4^−^ T cells (Figure S15D), or lung non-T (CD3^−^) lymphocytes (Figure S15E) were observed. Thus, lung epithelial antigen presentation functions in a very tissue-specific (i.e., lung but not blood) and CD4^+^ T cell-restricted fashion. Taken together, the results show that epithelial cell antigen presentation skews CD4^+^ T_RM_ cells to T_H_1 and T_H_2 biology and regulates airway hyperreactivity during neutrophilic allergic airway disease.

### Antigen presentation by epithelial cells regulates rapid allergic airway neutrophilia

**Figure 6.**
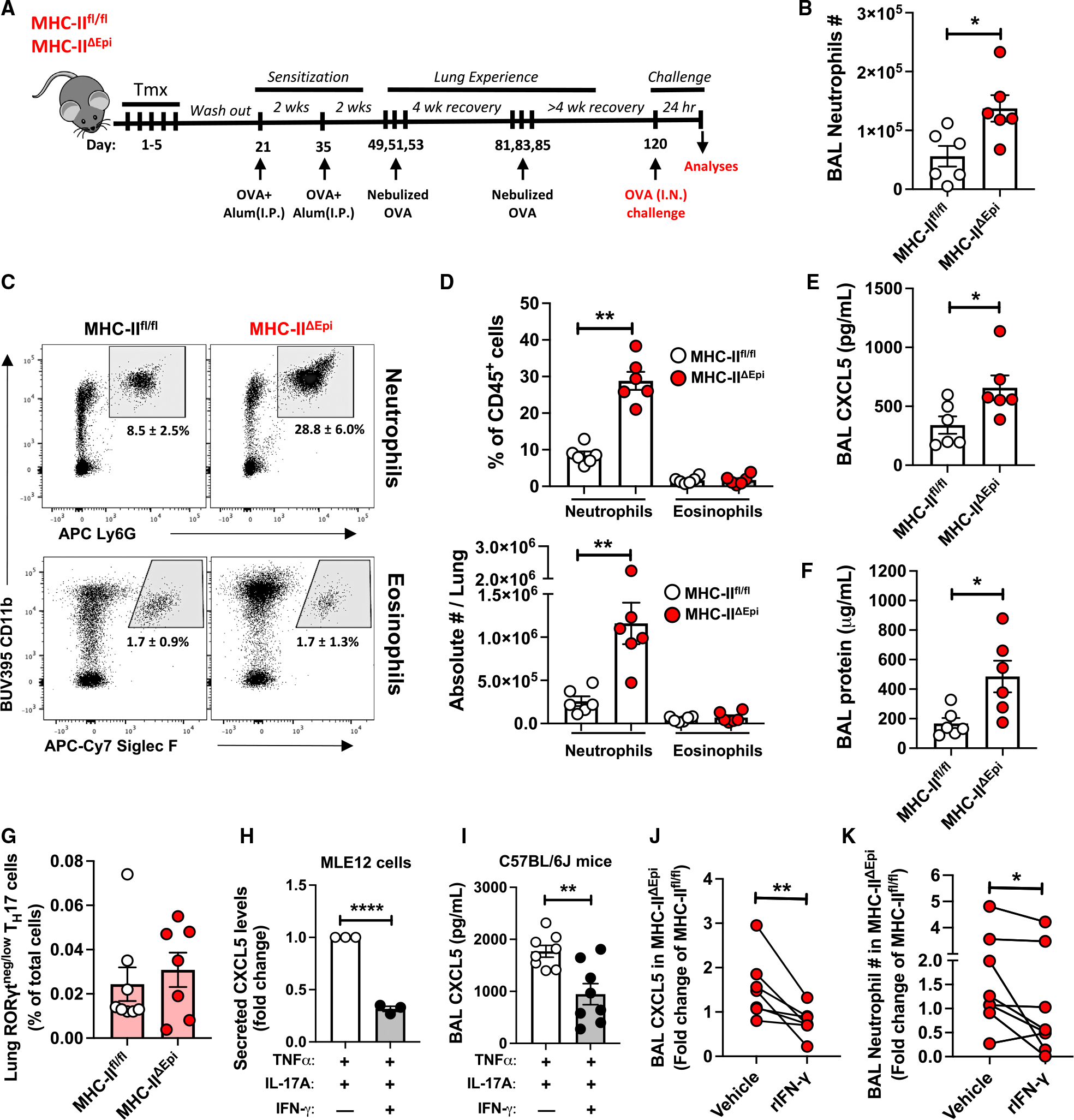
Lung epithelial MHC class II curtails severe allergic airway neutrophilia (A) Schematic of experimental timeline. (B) Total neutrophil numbers in bronchoalveolar lavages (BALs) from MHC class II^fl/fl^ and MHC class II^ΔEpi^ LH mice 24 post-OVA challenge. Mann-Whitney test. (C) Representative dot plots depicting lung (ivCD45.2^−^) neutrophils and eosinophils as fraction (%) of CD45^+^ cells 24 h post-OVA challenge; mean ± SD. (D) Numbers of lung (ivCD45.2^−^) neutrophils and eosinophils in MHC class II^fl/fl^ and MHC class II^ΔEpi^ LH mice 24 h post-OVA challenge. Mann-Whitney test. (E) BAL CXCL5 in MHC class II^fl/fl^ and MHC class II^Δepi^ LH mice 24 h post-OVA challenge. Mann-Whitney test. (F) Lung damage expressed as BAL protein content. Mann-Whitney test. (G) Relative frequency of RORγt^negative/low^ IL-17A^+^ lung (ivCD45.2^−^) CD4^+^ T cells in MHC class II^fl/fl^ and MHC class II^ΔEpi^ LH mice. Mann-Whitney test. (H) CXCL5 released by mouse lung epithelial (MLE12) cells treated with tumor necrosis factor alpha (TNF-α) plus IL-17A with or without IFN-γ for 6 h. Unpaired t test. (I) BAL CXCL5 in C57BL/6J mice 7 h post-administration of specified cytokine cocktails. Mann-Whitney test. (J and K) BAL CXCL5 (J) and BAL (K) neutrophil numbers in MHC class II^ΔEpi^ LH mice challenged with OVA plus vehicle or OVA plus IFN-γ 8 h post-challenge. Data are presented as the fold change over cage-mate MHC class II^fl/fl^ LH mice. Mann-Whitney test. *p ≤ 0.05 and **p ≤ 0.01. All data have *n* ≥ 4 mice, 2 experiments, and mean ± SEM.

Altered phenotypes of lung T_RM_ cells due to epithelial MHC class II ablation led us to question how the absence of epithelial MHC class II affects rapid allergic airway neutrophilia (Figure 6A). While mice of both genotypes possessed comparable BAL macrophage and lymphocyte numbers (Figures S15F–S15H), the inflamed MHC class II^ΔEpi^ mice showed greater neutrophil numbers within BAL (Figure 6B) and higher frequencies and numbers of extravascular (ivCD45.2^−^) neutrophils, but not eosinophils, in lungs after allergen challenge (Figures 6C and 6D). Furthermore, worsened neutrophilia was accompanied by elevated BAL CXCL5 levels (Figure 6E) and exacerbated airway edema (Figure 6F), despite comparable abundances of RORγt^negative/low^ T_H_17 T_RM_ cells (Figure 6G) and resulting IL-17A levels (Figure S15I) within MHC class II^ΔEpi^ lungs. Thus, lung epithelial MHC class II limits CXCL5 accumulation and neutrophilic inflammation in allergic airways by mechanisms distinct from, but seemingly downstream of, T_H_17 T_RM_ cells.

### T_H_1 cytokine IFN-γ curbs CXCL5 and the excess airway neutrophilia of MHC class II^ΔEpi^ mice

Because T_H_1 biology and its effector cytokine IFN-γ can inhibit T_H_17 cell responses^75^ and were curtailed by epithelial MHC class II deficiency (Figures 5E and 5F) alongside excess CXCL5 and neutrophils (Figures 6C–6E), we asked whether IFN-γ might influence CXCL5 expression downstream of IL-17A signaling in inflamed lung. Indeed, rIFN-γ blunted IL-17A-induced CXCL5 secretion by inflamed mouse lung epithelial cells *in vitro* (Figure 6H) and mouse airways *in vivo* (Figure 6I). Thus, IFN-γ from T_H_1 T_RM_ cells may prevent CXCL5 production and resultant neutrophilia driven by IL-17A in allergen-experienced airways. Conversely, diminished T_H_1 numbers (and hence reduced IFN-γ) due to epithelial MHC class II deficiency may lead to uncontrolled CXCL5 production and exacerbate neutrophil recruitment. If so, the excessive neutrophilia phenotype due to epithelial MHC class II deletion should be corrected by supplementing IFN-γ. Consistent with this, exogenous rIFN-γ (concomitant with allergen challenge) reduced CXCL5 levels (Figure 6J) and neutrophil influx (Figure 6K) in airways of allergic MHC class II^ΔEpi^ mice. Of note, the Muc5ac^high^ airway secretory epithelial cells that are predominant producers of CXCL6 in adult human airways also express receptors for IFN-γ (Figures S12G, S13H, and S14F),^14^ suggesting responsiveness to this T_H_1 cytokine and implicating this regulatory pathway in human lungs.

### IFN-γ inhibits allergic airway neutrophilia

**Figure 7.**
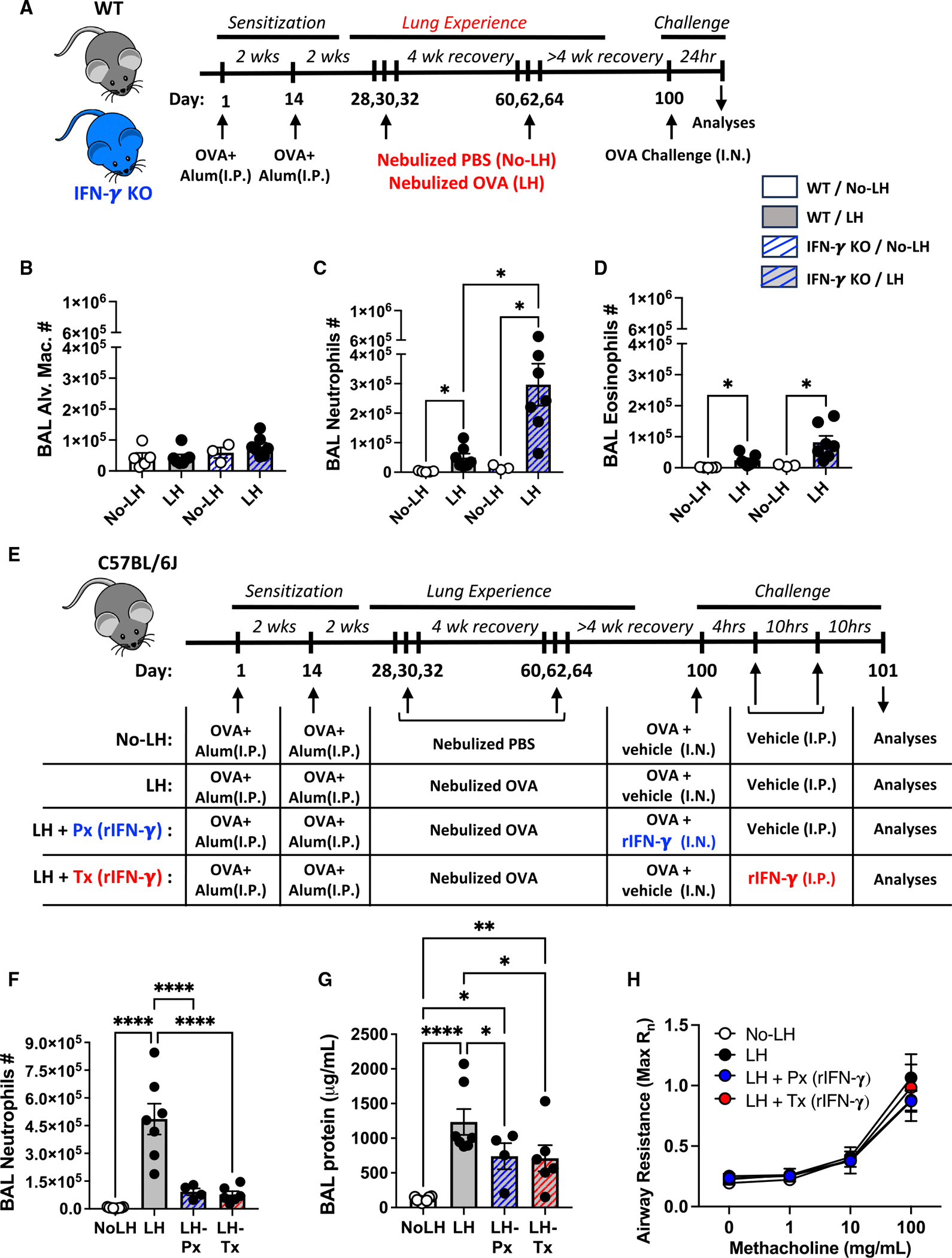
IFN-γ suppresses allergic airway neutrophilia (A) Schematic of experimental timeline. (B–D) Total numbers of alveolar macrophages (B), neutrophils (C), and eosinophils (D) in WT and IFN-γ-knockout no-LH and LH mice 24 h post-OVA challenge. One-way ANOVA with Fisher’s LSD test. Note: the y axes in (B)–(D) are adjusted to a similar scale to facilitate direct comparisons of airway inflammatory profiles. (E) Schematic of experimental timeline. (F) Total neutrophil numbers in BAL from mice 24 post-OVA challenge with specified treatment modalities. One-way ANOVA with Fisher’s LSD test. (G) Lung damage expressed as BAL protein content. One-way ANOVA with Fisher’s LSD test. (H) Airway reactivity in response to increasing doses of methacholine in mice 24 h post-OVA challenge with specified treatment modalities as measured by Flexivent. The measurements represent maximum Rn values. Two-way ANOVA with Fisher’s LSD test. *p ≤ 0.05, **p ≤ 0.01, ***p ≤ 0.001, and ****p ≤ 0.0001. All data have *n* ≥ 4 mice, 2–3 experiments, and mean ± SEM.

The ability of rIFN-γ to rescue excess neutrophilia in allergic MHC class II^ΔEpi^ mice suggested a finding of potential translational value if such a strategy could mitigate allergic neutrophilia more broadly. Given the lack of effective therapies for patients with neutrophilic asthma and the availability of rIFN-γ as an FDA-approved treatment for chronic granulomatous disease and severe malignant osteopetrosis,^76^ we explored IFN-γ as a potential opportunity to mitigate neutrophilic allergic airway disease. (1) Consistent with the immunoregulatory role played by IFN-γ during allergic airway neutrophilia, IFN-γ-knockout LH mice displayed unrestrained neutrophilic accumulation in the airways post-OVA challenge when compared to their no-LH counterparts (Figures 7A–7D). (2) We next asked if rIFN-γ delivered as a prophylactic (Px) prevented neutrophilic asthma exacerbations (Figure 7E, blue track). A single intranasal instillation of rIFN-γ delivered during the OVA challenge was sufficient to prevent allergic airway neutrophilia (Figure 7F) and resulting damage (Figure 7G) without affecting IL-17A levels in the LH lungs (Figure S16A). Although encouraging, patients with asthma experience sudden and unpredictable onsets of allergic asthma exacerbation. (3) Therefore, we also tested whether Tx delivery of rIFN-γ (delivered 4 h after inhaled OVA challenge, when sickness behavior due to acute asthma exacerbations is detectable in LH mice; Figure S1E) can limit the severity of neutrophilic asthma (Figure 7E, red track). The systemic administration of rIFN-γ potently suppressed allergic airway neutrophilia (Figure 7F) and airway edema (Figure 7G) without affecting IL-17A in the LH lungs (Figure S16A) when delivered as a Tx regimen. These suppressive effects of rIFN-γ on airway neutrophilia can be retrospectively confirmed in humans since nebulized or subcutaneously administered rIFN-γ robustly reduced BAL CXCL5 levels and neutrophils in airways of patients with idiopathic pulmonary fibrosis^77^ or pulmonary tuberculosis.^78^ Of note, despite favorable anti-neutrophilic outcomes, neither Px nor Tx rIFN-γ delivery improved airway hyper-reactivity in the acute setting of allergic asthma exacerbation tested in our studies (Figure 7H). However, since IFN-γ signaling blunts T_H_2 cell development and pathogenesis in asthma,^79–84^ it is plausible that repeated rIFN-γ therapy may gradually show positive outcomes with respect to airway physiology as well. Indeed, small, independent clinical studies in patients with steroid-resistant asthma revealed that recurrent systemic administration of rIFN-γ improved lung function and reduced the severity of asthma outcomes.^85,86^ Thus, our findings suggest that IFN-γ (and the pathways it triggers) could represent a rational, promising approach to mitigating neutrophilic asthma.

## DISCUSSION

Recurrent but transient exposures of sensitized mice to inhaled OVA seeds their lungs with a mature epithelial and immune ensemble that mirrors adult human lung biology, instills human predisposition to neutrophilic asthma, and thus provides a tractable animal model for the rapid allergic airway neutrophilia characteristic of this vexing disease. A notable feature of such experienced lungs includes the establishment of diverse clusters of tissue-resident CD4^+^ T_RM_ cells comprised of unconventional RORγt^negative/low^ T_H_17 cells. Reactivation of these CD4^+^ T_RM_ cells leads to the rapid secretion of IL-17A, inducing expression of CXCL5 by Muc5ac^high^ airway secretory epithelial cells, which elicit prompt peribronchial neutrophilic infiltration and severe disease. Antigen presentation by epithelial cells regulates disease severity by supporting protective T_H_1 T_RM_ cell activities, which curb the production of CXCL5 and neutrophilia driven by IL-17A in allergen-experienced airways. Thus, our studies identify key cellular and molecular constituents of biology relevant to human neutrophilic asthma, establishing a role for CD4^+^ T_RM_ cell-epithelial cell crosstalk in calibrating disease severity and identifying IFN-γ (and immunoregulatory pathways it triggers) as a promising Tx avenue against the disease with direct translational implications.

Based on the age of diagnoses, asthma can be broadly classified into two broad phenotypes: childhood asthma, which is dominated by T_H_2 cell-driven, steroid-sensitive eosinophilic inflammation, and late-onset asthma, associated with lung-damaging, steroid-refractory neutrophilic inflammation.^1,3,5,8^ A subset of patients transition from having treatment-responsive disease to becoming treatment resistant over time.^9–12^ Pathways leading to these differential outcomes in an age-dependent manner need further understanding and study. Our work suggests that T_H_2-high eosinophilic and “T_H_2-low” neutrophilic endotypes of asthma may not be separate diseases but rather represent extremities of a continual spectrum (Figure S17A). The progression in asthma endotypes may thus be linked to the frequency and extent of aeroallergen exposure that occurs with time and advancing age. Specific elements of aeroallergenic experience may differentiate the progression of eosinophilic asthma to neutrophilic disease from the induction of antigenic tolerance, including the frequencies in durations of exposure.^49^

Our studies identify a previously unrecognized diversity within the CD4^+^ T_RM_ cell pool residing in lungs prone to neutrophilic asthma. CD4^+^ T_RM_ cells within allergen-experienced murine and human lungs have been reported to be of T_H_2 or Treg T_RM_ cell phenotypes.^13,15–19,87^ The present studies extend roles for CD4^+^ T_RM_ cells to neutrophilic asthma and show that allergic lungs can harbor diverse T_RM_ cell types, including Treg, T_H_1, T_H_2, and T_H_17 T_RM_ cells, among a yet larger pool of T_RM_ lymphocytes that are negative for all the major LDTFs often tested. CD4^+^ T_RM_ cells in lungs with inhaled allergen history could produce IL-17A with minimal to no expression of the T_H_17-defining transcription factor RORγt. Although surprising, inspection of a scRNA-seq study profiling airway and peripheral blood CD4^+^ T cells of adult asthmatic and healthy humans confirmed the T cell heterogeneity observed in mice, including the unconventional RORγt^negative/low^ T_H_17 CD4^+^ T_RM_ cells,^14,70^ providing human disease correlates to our murine discoveries. Independent of the lungs or allergies, the population of T_H_17 cells recently described as pathogenic in a mouse model of multiple sclerosis^88^ also exhibited minimal RORγt expression compared to their homeostatic T_H_17 counterparts, extending the significance of such unconventional T_H_17 cells to diverse allergic and autoimmune diseases of human health relevance. Further studies are warranted to determine whether these poorly defined pathogenic T_H_17 cells may be amenable to pharmacologic intervention.

Lung epithelial cells display anatomically segregated abilities to communicate with CD4^+^ T cells and neutrophils during allergic airway neutrophilia (Figure S17B). Constitutive MHC class II expression on alveolar type 2 (AT2) epithelial cells has been reported before,^89,90^ and AT2 MHC class II was described to induce tolerance to inhaled allergens in a mouse model of eosinophilic asthma.^71^ Whether epithelial antigen presentation may be involved in neutrophilic asthma was unclear. Here, we find Muc5ac^high^ secretory cells in the airways to be an especially immunorelevant cell type. This was reflected in their high expression of antigen presentation molecules and simultaneous elaboration of CXCL5 (or CXCL6 in humans) within experienced airways. In the setting of pneumococcal pneumonia, T_H_17 T_RM_ cells, via secretion of IL-17A, instigate enhanced secretion of CXCL5 from lung epithelial cells to enforce rapid neutrophil recruitment and anti-bacterial immunity.^41,63^ Our current study extends this cell signaling axis to allergic airway neutrophilia and suggests that CXCL6 (the human ortholog of murine CXCL5) might be a good biomarker for neutrophilic asthma. CXCL6 correlates with disease severity in asthmatics.^91–93^ The observation that secretory cells are select sources of murine CXCL5 or human CXCL6 in neutrophilic allergic airway disease, in conjunction with a previous report demonstrating that depletion of airway secretory cells reduces T_H_2 cell responses, eotaxin production, and eosinophil recruitment during eosinophilic asthma,^94^ highlights these cells as consistently instrumental to both T cells and granulocytes in the allergic lung. CD45^−^EpCAM^−^ stromal cells were additional sources of CXCL5 in neutrophilic allergic airways, bolstering the expanding knowledge about immunological functions for stromal cells during lung diseases.^95–99^ Which cell types within the structural cell fraction are CXCL5 producers is unclear but may include peribronchial fibroblasts, airway smooth muscle cells, pericytes, and/or endothelial cells.^73,100–104^

Epithelial antigen presentation regulates the severity of allergic airway neutrophilia by instructing CD4^+^ T_RM_ cell activities. While epithelial MHC class II supported the formation of T_H_2 T_RM_ cells and bolstered airway hyper-reactivity, it also enriched the lung T_RM_ pool for T_H_1 T_RM_ cells that appeared to curb allergic airway neutrophilia. Thus, lung epithelial antigen presentation functions as a rheostat that sets the fine balance between two clinical symptoms of neutrophilic asthma: airway hyper-reactivity and rapid allergic airway neutrophilia. It does so by altering the balance of T_RM_ cells and reinforcing protective T_H_1 cell activities in the airways (Figure S17B). Elevated T_H_1 cell numbers are observed in asthmatic lungs^105,106^ and may reflect an attempt of the inflamed airways to limit allergic inflammation, implicating T_H_1 cells as mitigating agents rather than pathogenic drivers in this allergic disease. Given that MHC class II on epithelial cells is key to airway T_RM_ cell biology, the polymorphisms in MHC class II-related genes that have been linked with adult-onset asthma^107,108^ may be significant for influencing the relative frequencies and activities of CD4^+^ T_RM_ cell lineages in the airways.

Expanding beyond its established roles in stifling T_H_2-high eosinophilic asthma,^79–84^ we now surmise that T_H_1 cytokine IFN-γ can regulate pathogenic T_H_17-driven neutrophilic asthma. However, rather than via the previously reported direct inhibition of T_H_17 cell differentiation and activities by IFN-γ,^75^ the protective function of T_H_1 activities during neutrophilic asthma is exerted downstream of the T_H_17 cells by IFN-γ blunting IL-17A-induced CXCL5 production in epithelial cells of the allergic airways. This defines a novel pathway for the regulation of type 17 inflammation by IFN-γ without the direct inhibition of T_H_17 cells. Whether IFN-γ suppresses *de novo* CXCL5 transcription or uncouples the IL-17A-mediated stabilization of CXCL5 mRNA is under active investigation.^41,109^ Nevertheless, our discovery of the suppressive effects of IFN-γ on airway neutrophilia and lung damage suggests this T_H_1 effector cytokine and the pathways it triggers as promising avenues for future research, with potential translational and clinical utility for neutrophilic asthma. This is also supported by an empirical clinical study.^85^

Taken together, our studies, using an OVA-induced mouse model of allergic airway disease, suggest that asthma endotypes may represent stages in a continuum of disease progression and offer insights into mechanisms underlying the pathophysiology of neutrophilic asthma. Our results implicate a T_H_17-CXCL5 axis in rapid allergic airway neutrophilia, with IL-17A in such lungs coming from unusual RORγt^negative/low^ CD4^+^ T cells. We define lung epithelial cells as key regulators and pathogenic effectors in the immune circuitry programmed within allergen-experienced airways of mice and humans, owing to their ability to instruct CD4^+^ T_RM_ cell activities (via antigen presentation) and direct airway neutrophilia (by acting as signaling nodes that integrate cues from pathogenic T_H_17 T_RM_ cells and T_H_1 T_RM_ cells to effectively fine-tune CXCL5 release). These studies advance the concept that IFN-γ can curb the effects of IL-17A on epithelial cells (including specifically their CXCL5 expression), leading to studies that now suggest that IFN-γ and/or the pathways this cytokine triggers deserve further investigation as promising Tx avenues for mitigating neutrophilic asthma.

### Limitations of the study

To focus selectively on how inhaled allergen experience may remodel the lung, we chose OVA instead of a clinically relevant but complex and less-defined allergen preparations of house dust mites (HDMs), cockroaches, or fungi. This was in order to minimize the contributions of confounding factors like trained immunity (or other off-target immune-conditioning events) and allow precise and effective determination of the effects of inhaled allergen experiences on disease outcomes in sensitized hosts. This, however, limits translation because OVA is not an asthma-relevant allergen. Also, the mice in our study were initially sensitized to OVA with alum as an adjuvant via the peritoneum, which is not akin to the human experience, where allergen exposure mostly occurs at the mucosal site. Thus, the first exposure of T cell-instructive DCs to allergen occurred via peritoneal DCs rather than those from the airways, which may have different phenotypes. Finally, while rIFN-γ treatment was sufficient to reduce the severity of allergic airway disease in our reductionist mouse model as well as some steroid-resistant human asthmatics,^85,86^ T_H_1 cells and IFN-γ can drive neutrophilic airway inflammation in other conditions.^110–112^ Thus, we emphasize caution and the need for further preclinical studies before considering IFN-γ as a potential means of limiting neutrophilic asthma. Instead, our findings direct us toward exploring the signaling pathways triggered by IFN-γ in this and other preclinical models of asthma to see if translational and clinical opportunities against neutrophilic asthma may arise by studying this immunoregulatory biology more carefully.

## RESOURCE AVAILABILITY

### Lead contact

Correspondence, further information, and requests for resources and reagents should be directed to and will be fulfilled by the lead contact, Anukul T. Shenoy (anukuls@umich.edu). All data will be made available by the lead contact upon reasonable request.

### Materials availability

This study did not generate new unique reagents.

### Data and code availability

- All data reported in this paper will be shared by the lead contact upon request.
- This paper does not report original code.
- Any additional information required to reanalyze the data reported in this paper is available from the lead contact upon request.

## STAR★METHODS

### EXPERIMENTAL MODELS AND STUDY PARTICIPANT DETAILS

6-week-old C57BL/6J (Stock# 000664), IL-17A/F knockout (B6.Cg-Il17a/Il17f^tm1.1Impr^ Thy1^a^/J, Stock # 034140),^113^ and IFN-γ knockout (B6.129S7-Ifng^tm1Ts^/J, Stock# 002287)^114^ mice were obtained from Jackson labs (USA). SPC-GFP mice and Nkx2.1^cre/ERT2^H2-Ab1^fl/fl^ mice (henceforth called MHCII^ΔEpi^ mice) are described elsewhere^69,71^ and were bred in-house. All breeders were maintained as homozygous floxed for H2-Ab1. For experiments all mice were homozygous for *loxP* sites flanking H2-Ab1 exon1 and were identified as Cre-positive or negative based on presence or absence of Nkx2.1^cre/ERT2^. Cage and littermate controls of both sexes were used for studies. 7–14-week-old mice were used for experiments and animals were housed in specific pathogen free environment on a 12-h light-dark cycle, with *ad libitum* access to standard chow and water. Mice were euthanized using isoflurane overdose and death confirmed using pneumothorax before organ collections. All animal procedures were approved by the Institutional Animal Care and Use Committee at the University of Michigan at Ann Arbor and Boston University.

### METHOD DETAILS

#### Experimental allergic airways eosinophilic disease

OVA (Sigma Aldrich) or sterile PBS was adsorbed on Alum (Sigma Aldrich) for 30 min on shaker before sensitization of mice to OVA at dose of 25μg/100μL via intraperitoneal (i.p.) injection of 100μL on days 1 and 14. OVA or PBS sensitized mice were then exposed to nebulized solution of 2% OVA in sterile PBS for 30 min in a nebulization chamber on days 28, 29 and 30. On day 31, mice were anesthetized with ketamine and xylazine before receiving intranasal (I.N.) challenge with 100μL of 0.2% OVA in sterile PBS.

#### Experimental allergic airways neutrophilic disease

OVA (Sigma Aldrich) or sterile PBS was adsorbed on Alum (Sigma Aldrich) for 30 min on shaker before sensitization of mice to OVA at dose of 25μg/100μL via intraperitoneal (i.p.) injection of 100μL on days 1 and 14. OVA sensitized mice were then split into two groups and exposed to nebulized solution of 2% OVA in sterile PBS or plain sterile PBS (for control mice) for 30 min in a nebulization chamber on days 28, 30, 32 and days 60, 62, 64 with a 4 week recovery period in between. After at least 4 weeks or more recovery period, mice were then anaesthesized with ketamine and xylazine before receiving intranasal (I.N.) challenge with 100μL of 0.2% OVA in sterile PBS.

#### Pneumococcus infections

*Streptococcus pneumoniae* (*Spn*)-specific lung-resident CD4^+^ T_RM_ cells were generated as previously described.^41,63^ Briefly, mice were intratracheally infected with ~10^6^ CFU of serotype 19F *Spn* (Strain EF3030) suspended in sterile saline on days 0 and 7 followed by recovery for 28–35 days. For pneumococcal infections to test antigen specificity of airway neutrophilia, mice were intratracheally infected with ~10^6^ CFU of serotype 3 *Spn* (Sp3, ATCC 6303) suspended in sterile saline as previously described.^41^

#### Physiological measurements of airway hyper-reactivity

For airway resistance assay, mice were injected with xylazine (10 mg/kg body weight), pentobarbital (100 mg/kg body weight) and pancuronium (0.5 mg/kg body weight), intubated and placed on a mechanical ventilator (Legacy flexiVent, SCIREQ). Ventilation was at 300 breaths/min (tidal volume 6–7 mL/kg body weight). Airway resistance was measured after airway delivery of nebulized methacholine in PBS (0, 1, 10, and 100 mg/mL). The plotted measurements represent Maximum Newtonian resistance (Rn) values.

#### Tamoxifen, dexamethasone, antibody administration

Tamoxifen (Sigma) was dissolved in corn oil (Sigma) to 20 mg/mL stock concentration and stored at 4°C. Mice were i.p. injected at 100 mg/kg of body weight for 5 consecutive days followed by a washout of at least 2 weeks before experimentation. Dexamethasone (1 mg/kg body weight) was delivered intraperitoneally 72 and 24 h prior to the OVA challenge to assess steroid responsiveness of the mice. CD4^+^ T_RM_ cells were depleted as described previously.^41^ Briefly, mice were administered 500μg and 100μg of GK1.5 (BioXcell, West Lebanon, NH) i.p. and i.n. respectively, both 72 and 24 h prior to the OVA challenge.

#### Lung histology

Euthanized mice were exsanguinated, and their tracheas cannulated with 25 gauge butterfly needle before inflation of lungs with 4% paraformaldehyde at 23 cm H_2_O pressure. The left lobes were paraffin embedded after overnight fixation in 4% paraformaldehyde, cut into 5μm thin sections and stained using hematoxylin and eosin.

#### Bronchoalveolar lavage (BAL) collection and analyses

Euthanized mice were exsanguinated, and their tracheas cannulated with a 18 gauge cannula followed by 6 rounds of lavage with sterile PBS. The supernatant from first lavage was stored at −80°C for BAL chemokine and protein measurements while the cell pellets from all lavages per mouse were compiled to perform flow cytometry or cytospins, differential staining and airway cellularity enumeration. BAL proteins levels were measured using standard BCA assay. BAL CXCL1, CXCL2, CXCL5 and CXCL10 levels were measured using ELISA kits from R&D Systems using the manufacturer’s protocols.

#### Lung digestion for flow cytometry

To enumerate of extravascular versus intravascular fraction of lung CD4^+^ T cells and myeloid cells, anesthetized mice were retroorbitally administered 2μg anti-CD45.2 antibody 3–5 min prior to euthanasia.^115^ Lungs were collected in RPMI 1640 with 10% FBS for flow cytometry. Single-cell suspensions were prepared by digestion of lungs as previously described by *Smith* et al.^63^ For high throughput flow cytometry and FACS sorting of lung epithelial cells, single cell suspensions of lungs were generated as previously described by *Shenoy* et al.^69^ Cells in single cell suspensions were blocked with TruStain αCD16/CD32 Fc-Block (BioLegend). Flow cytometry was performed on LSR Fortessa or LSR II Flow Cytometer (both BD Biosciences) and data was analyzed with FlowJo software (BD Biosciences). High-dimensional multi-parameter spectral flow cytometry was performed on Aurora (Cytek). SpectraFlo (Cytek) software was utilized for spectral unmixing of the data using ordinary least square algorithm and and data were analyzed using FlowJo (BD Biosciences). Gating strategies are provided in the Supplemental figures and were based on use of Fluorescence minus one (FMO) controls.

For RNA-profiling, leukocytic (CD45^+^), epithelial (CD45^−^EpCAM^+^) and stromal (CD45^−^EpCAM^−^) cell fractions from stained single cell suspensions were sorted into RLT buffer with beta-mercaptoethanol on ice using FACS-Aria II SORP (BD Biosciences) before proceeding to RNA extraction.

#### *Ex vivo* stimulation of CD4^+^ T cells

To allow identification of extravascular versus intravascular fraction of lung CD4^+^ T cells, anesthetized mice were retro-orbitally administered 2μg anti-CD45 antibody 3–5 min prior to euthanasia. Single cell suspension of lung leukocytes was prepared as described before and 2 × 10^6^ cells were stimulated *ex vivo* in 12 well plate with 250 ng/mL Phorbol Myristate Acetate (PMA)(Sigma Aldrich) and 1.5 μg/mL Ionomycin (Sigma, St. Louis, MO) in T cell stimulation media for 1hour at 37°C and 5% CO_2_. Monensin (Biolegend, Cat# 420701) and Brefeldin A (Biolegend, Cat# 420601) both at 1X final concentration were added to the cell suspension for last 5 h at 37°C and 5% CO_2_. Cells were then processed for intracellular cytokine and transcription factor staining, as per manufacturer’s protocols.

#### Intracellular cytokine, protein and transcription factor staining for flow cytometry

For intracellular staining of transcription factors and Ki67, eBioscience Foxp3/Transcription Factor Staining Buffer Set (Cat# 00–5523-00) was used as per manufacturer’s protocols. For intracellular cytokine staining, eBioscience Intracellular Fixation & Permeabilization Buffer Set (Cat# 88–8824-00) was used as per manufacturer’s protocols. For intracellular costaining of cytokines and transcription factors, eBioscience Foxp3/Transcription Factor Staining Buffer Set (Cat# 00–5523-00) was used.

#### Algorithmic analysis of single cell fluorescence datasets

Data processing pipeline was established using the Omiq.ai cloud computation platform (Omiq) as previously described.^69^ Briefly, live CD45^+^CD4^+^CD19^−^CD45.2^+^ for lung intravascular (‘blood’) cells or live CD45^+^CD4^+^CD19^−^CD45.2^−^ for lung extravascular (‘lung’) T cells were concatenated, asinh transformed (cofactor = 6000) and clustered with Phenograph algorithm (k = 20, distance metric = euclidean)^67,68^ followed by prejection in opt-SNE space (perplexity = 30, theta = 0.5, opt-SNE endpoint = 5000; PCA pre-initialization embedding). For data in Figure 5, CD62L^−^CD44^+^CD11a^+^ clusters (as determined based on the MFI cutoff) were subsampled as memory T cell data. These data were then re-clustered with Phenograph (k = 20, distance metric = euclidean) and projected into opt-SNE space (perplexity = 30, theta = 0.5, opt-SNE endpoint = 5000; PCA pre-initialization embedding). Clusters were color-coded and overlaid on the opt-SNE projections. Each marker MFIs of Phenograph clustered datasets were organized into hierarchically clustered heatmaps. Clusters were classified as positive for specific lineage-defining transcription factors (LDTF) based on the MFI value cut offs set by measuring the MFIs of LDTF-negative non-T cell populations sampled from the same dataset. This approach was preferred over the FMO-based cutoff calculation to alleviate the MFI difference caused by non-specific binding of anti-LDTF antibodies to cells that do not express them. Frequencies of each cell type and MFI values for individual markers were calculated from corresponding clusters per each animal and data were plotted and compared using Prism 8.0 (Graphpad).

#### Analyses of independently generated adult human lung single cell RNA-Seq datasets

Previously published single cell RNA-Seq datasets profiling epithelial cells and CD4^+^ T cells from blood and lungs of adult humans and asthmatics were interrogated for specified target transcripts using their respective interactive webtools.^14,73^ For reanalyses of human airways dataset of adult asthmatic and healthy controls from Alladina Sci. Immunol. 2023,^70^ processed data was obtained from the GEO database (under accession number GSE193816). UMAP plots were generated using the *pl.umap* function in Scanpy^116^ (version 1.9.4). Dot plot was created using *pl.scatter*. Threshold lines were defined based on the mean gene expression values. For reanalyses of murine allergic airway eosinophilia dataset from Tibbitt et al. Immunity 2019,^17^ raw data was downloaded from the GEO database (under accession number GSE131935). The raw count matrices were processed using Scanpy^116^ (version 1.9.4), following a standard pipeline: *pp.normalize_total, pp.log1p, pp.highly_variable_genes, tl.pca, pp.neighbors, tl.leiden,* and *tl.umap*, all with default parameters. UMAP plots were generated using the *pl.umap* function, and dot plot was created with *pl.scatter*. As with the human dataset, threshold lines were set based on the mean gene expression values.

#### Immunofluorescent staining

The trachea of euthanized mice were cannulated with an 18-gauge angiocath and 1.4 mL of Tissue-Tek Optimal Cutting Temperature (O.C.T.) compound (Sakura Finetek) was slowly instilled in the lung. Once the lungs were inflated, the left bronchus was tied using a suture, the lungs washed in sterile HBSS before embedding in cryomolds with O.C.T. and flash freezing at −80°C until sectioning. Frozen 8 μm thin coronal sections of the left lungs that contained the whole face of the lungs including the entire airway tree structure were collected for further analyses. Sections with folds and/or tears were rejected. The sections that met our inclusion criteria were first fixed, washed, and permeabilized (with 0.2% Triton X-) before blocking with Blocking buffer (PBS with 10% normal donkey serum and 3% BSA) followed by overnight incubation with rabbit antimouse CD4 (abcam, Cat# ab183685) and rat antimouse Ly-6G (clone 1A8; BD Biosciences) at 4°C in a humidified chamber. Next day, sections were vigorously washed before incubation with Alexa 594 conjugated Affinipure donkey antirabbit IgG and Alexa 488 conjugated Affinipure donkey antirat IgG (Jackson Immunoresearch) at room temperature for 1 h in a dark humidified chamber. All slides were counterstained with DAPI (Molecular Probes by Life Technologies, R37606) before mounting the sections with FluorSave (Millipore Calbiochem: 345789) and covering with coverslip for visualization. Of note, “no primary antibody control” were used to identify true events. Images were captured using a Leica DM4 microscope equipped with Leica DFC 7000T camera and processed using ImageJ 2.0.0-rc-69.

#### *In vitro* studies on lung epithelial cell line

Mouse lung epithelial (MLE12) cells were treated with cell culture media containing TNF-α (25 ng/mL) + IL-17A (50 ng/mL) with or without IFN-γ (50 ng/mL) for 6 h before collection of supernatant for CXCL5 ELISA. All mouse cytokines were purchased from R&D Systems. The MLE12 cell line we used is not authenticated but were routinely tested and found to be negative for mycoplasma contamination.

#### RNA extraction and real-time PCR

RNA was extracted from sorted cells using RNAeasy Micro Kit (Cat# 74004) as per manufacturer’s protocols and stored at −80°C. qRT-PCR was performed using the RNA-to-Ct kit (Life Technologies). Commercially available TaqMan gene expression assay primers and probes for cxcl1, cxcl2, cxcl5, cxcl10, muc5ac and 18S rRNA (Cat# 4319413E) from Applied Biosystems were used. The quantity of the detectable mRNA was calculated by normalizing to 18S rRNA from the respective sample and then expressed as fold change over mRNA levels of whole lung of naive mice.

#### *In vivo* recombinant IFN-γ administration

Mice were administered 100ng of recombinant mouse IFN-γ (or vehicle) in 100μL volume for intranasal Px-rIFN-γ administrations and 200ng of recombinant mouse IFN-γ (or vehicle) in 200μL volume for intraperitoneal Tx-rIFN-γ administrations at designated time-points. Recombinant mouse IFN-γ was purchased from R&D Systems.

### QUANTIFICATION AND STATISTICAL ANALYSES

Statistical analyses were performed using Prism 10 (Version 10.3.0, GraphPad). Differences were deemed statistically significant if the *p* value or FDR *q* value was ≤0.05. Each figure legend communicates the number of mice used, the experiment replicates performed and the statistical tests used to make comparisons. For all figures, data are represented as mean ± SEM, except for Figures 1G and 6C which depicts flow cytometry plots with data represented as mean ± SD).

## Supplementary Material

1

## Figures and Tables

**KEY RESOURCES TABLE:** 

REAGENT or RESOURCE	SOURCE	IDENTIFIER

Antibodies		

BUV737 anti-mouse CD45.2 (Clone 104)	BD Biosciences	Cat# 612778; RRID: AB_2870107
APC-Cy7 anti-mouse EpCAM (Clone G8.8)	Biolegend	Cat# 118218; RRID: AB_2098648
PerCP Cy5.5 anti-mouse I-A/I-E (MHC-II, Clone M5/114.15.2)	BD Biosciences	Cat# 562363; RRID: AB_11153297
PE anti-mouse CD40 (Clone 3/23)	BD Biosciences	Cat# 553791; RRID: AB_395055
PE-Cy7 anti-mouse PD-L1 (Clone 10F.9G2)	Biolegend	Cat# 124314; RRID: AB_10643573
PE anti-mouse PD-L1 (Clone 10F.9G2)	Biolegend	Cat# 124308; RRID: AB_2073556
BV421 anti-mouse CD24 (Clone M1/69)	BD Biosciences	Cat# 562563; RRID: AB_2737002
PE Cy7 anti-mouse CD24 (Clone M1/69)	BD Biosciences	Cat# 560536; RRID: AB_1727452
APC anti-mouse CD104 (Clone 346–11A)	Biolegend	Cat# 123612; RRID: AB_2734182
SB600 anti-mouse Podoplanin (Clone 8.1.1)	Invitrogen	Cat# 63–5381-82; RRID: AB_2735074
Alexa Flour 700 anti-mouse I-A/I-E (MHC-II, Clone M5/114.15.2)	Biolegend	Cat# 107621; RRID: AB_493726
Alexa Flour 532 anti-mouse CD45 (Clone 30-F11)	Invitrogen	Cat# 58–0451-82; RRID: AB_11218871
BV510 anti-mouse CD54/ICAM1 (Clone 3E2)	BD Biosciences	Cat# 563628; RRID: AB_2738331
PE Dazzle594 anti-mouse CD80 (Clone 16–10A1)	Biolegend	Cat# 104737; RRID: AB_2564174
BV650 anti-mouse CD86 (Clone GL-1)	Biolegend	Cat# 105035; RRID: AB_11126147
BV480 anti-mouse PD-L2 (Clone TY-25)	BD Biosciences	Cat# 746756; RRID: AB_2744017
BV510 anti-mouse CD45 (Clone HI30)	Biolegend	Cat# 304036; RRID: AB_2561940
FITC anti-mouse EpCAM (Clone 9C4)	Biolegend	Cat# 324204; RRID: AB_756078
PE Cy7 anti-mouse PD-L1 (Clone 29E.2A3)	Biolegend	Cat# 329718; RRID: AB_2561687
BV510 anti-mouse PD-1 (Clone 29F. 1A12)	Biolegend	Cat# 135241; RRID: AB_2715761
BV570 anti-mouse CD44 (Clone IM7)	Biolegend	Cat# 103037; RRID: AB_10900641
BV605 anti-mouse CD19 (Clone 6DS)	Biolegend	Cat# 115539; RRID: AB_11203538
BV650 anti-mouse CD62L (Clone MEL-14)	BD Biosciences	Cat# 564108; RRID: AB_2738597
BV786 anti-mouse CD11a (Clone M17/4)	BD Biosciences	Cat# 740866; RRID: AB_2740518
PE anti-mouse CD69 (Clone H1.2F3)	Biolegend	Cat# 104508; RRID: AB_313111
PE Cy5.5 anti-mouse CD25 (Clone PC61.5)	Invitrogen	Cat# 35–0251-80; RRID: AB_11218285
Alexa Fluor 647 anti-mouse CD3e (Clone 145–2C11)	Biolegend	Cat# 100322; RRID: AB_389322
Alexa Fluor 700 anti-mouse CD4 (Clone RM4–4)	Biolegend	Cat# 116021; RRID: AB_2715957
Alexa Fluor 488 anti-mouse CD8a (Clone 53–6.7)	Biolegend	Cat# 100723; RRID: AB_389304
BUV395 anti-mouse CD3 (Clone 145–2C11)	BD Biosciences	Cat# 563565; RRID: AB_2738278
BUV805 anti-mouse CD8a (Clone 53–6.7)	BD Biosciences	Cat# 612898; RRID: AB_2870186
eFluor450 anti-mouse Ki67 (Clone SolA15)	Invitrogen	Cat# 48–5698-80; RRID: AB_11151155
BV711 anti-mouse CD103 (Clone 2E7)	Biolegend	Cat# 121435; RRID: AB_2686970
FITC anti-mouse FOXP3 (Clone FJK-16s)	Invitrogen	Cat# 11–5773-82; RRID: AB_465243
PE-e610 anti-mouse GATA-3 (Clone TWAJ)	Invitrogen	Cat# 61–9966-41; RRID: AB_2574685
PE Cy7 anti-mouse T-bet (Clone 4B10)	Biolegend	Cat# 644823; RRID: AB_2561760
APC anti-mouse RORyt (Clone B2D)	Invitrogen	Cat# 17–6981-80; RRID: AB_2573253
Alexa Fluor 488 anti-mouse CD45 (Clone 30-F11)	Biolegend	Cat# 103122; RRID: AB_493531
BV510 anti-mouse CD4 (Clone GK1.5)	Biolegend	Cat# 100449; RRID: AB_2564587
APC anti-mouse CD11a (Clone M17/4)	Invitrogen	Cat# 17–0111-82; RRID: AB_11217471
BV421 anti-mouse CD44 (Clone IM7)	BD Biosciences	Cat# 563970; RRID: AB_2738517
PE Cy7 anti-mouse CD62L (Clone MEL-14)	Biolegend	Cat# 104418; RRID: AB_313103
APC Cy7 anti-mouse CD8a (Clone 53–6.7)	Biolegend	Cat# 100714; RRID: AB_312753
PerCP Cy5.5 anti-mouse CD103 (Clone 2E7)	Biolegend	Cat# 121416; RRID: AB_2128621
BV605 anti-mouse CD4 (Clone GK1.5)	Biolegend	Cat# 100451; RRID: AB_2564591
PerCP Cy5.5 anti-mouse CD45 (Clone 30-F11)	Biolegend	Cat# 103132; RRID: AB_893340
PE Cy7 anti-mouse CD3e (Clone 145–2C11)	Biolegend	Cat# 100320; RRID: AB_312685
FITC anti-mouse IL-17A (Clone TC11–18H10.1)	Biolegend	Cat# 506908; RRID: AB_536010
APC anti-mouse IL-5 (Clone TRFK5)	Biolegend	Cat# 504306; RRID: AB_315330
PE anti-mouse IL-13 (Clone eBio13A)	Invitrogen	Cat# 12–7133-41; RRID: AB_10852712
APC Cy7 anti-mouse IFN-y (Clone XMG1.2)	Biolegend	Cat# 505850; RRID: AB_2616698
BV421 anti-mouse IL-4 (Clone 11B11)	Biolegend	Cat# 504120; RRID: AB_2562102
PE Cy7 anti-mouse CD11c (Clone HL3)	BD Biosciences	Cat# 558079; RRID: AB_647251
eFluor450 anti-mouse Ly-6C (Clone HK1.4)	Invitrogen	Cat# 48–5932-82; RRID: AB_10805519
PE anti-mouse CD103 (Clone 2E7)	Biolegend	Cat# 121406; RRID: AB_1133989
BV510 anti-mouse CD45 (Clone 30-F11)	BD Biosciences	Cat# 563891; RRID: AB_2734134
FITC anti-mouse CD64 (Clone X54–5/7.1)	Biolegend	Cat# 139316; RRID: AB_2566556
APC Cy7 anti-mouse Siglec-F (Clone E50–2440)	BD Biosciences	Cat# 565527; RRID: AB_2732831
APC anti-mouse Ly-6G (Clone 1A8)	BD Biosciences	Cat# 560599; RRID: AB_1727560
BUV395 anti-mouse CD11b (Clone M1/70)	BD Biosciences	Cat# 563553; RRID: AB_2738276
Pure anti-mouse CD16/32 Fc Block (Clone 93)	Biolegend	Cat# 101302; RRID: AB_312801
Rabbit anti-mouse CD4	Abcam	Cat# ab183685; RRID: AB_2686917
Rat anti-mouse Ly-6G (Clone 1A8)	BD Biosciences	Cat# 551459; RRID: AB_394206
BV650 anti-mouse CD45.2 (Clone 104)	Biolegend	Cat# 109835; RRID: AB_11203374
BV650 Rat Anti-Mouse I-A/I-E (Clone M5/114.15.2)	Biolegend	Cat# 107641; RRID: AB_2565975
BUV395 anti-mouse PD-L2 (Clone MIH37)	BD Biosciences	Cat# 752604; RRID: AB_2917591
BV786 anti-mouse CD86 (Clone GL1)	Invitrogen	Cat# 417–0862-80; RRID: AB_3074143
BUV737 anti-mouse CD80 (Clone 16–10A1)	BD Biosciences	Cat# 612773; RRID: AB_2870102
BV786 anti-mouse RORyt (Clone Q31–378)	BD Biosciences	Cat# 564723; RRID: AB_2738916
7-aminoactinomycin D (7-AAD)	Invitrogen	Cat# A1310
eFluor 450 anti-mouse Ly6G (Clone 1A8-Ly6g)	Invitrogen	Cat# 48–9668-82; RRID: AB_2637124
PerCP-Cy5.5 anti-mouse CD45.2 (Clone 104)	Biolegend	Cat# 109828; RRID: AB_893350
PE-eFluor610 anti-mouse Gata3 (Clone TWAJ)	Invitrogen	Cat# 61–9966-42; RRID: AB_2574686
Alexa Fluor 488 anti-mouse CD45 (Clone 30-F11)	Invitrogen	Cat# 103122; RRID: AB_493531
InVivoMAb anti-mouse CD4 (Clone GK1.5)	BioXCell	Cat# BE0003–1; RRID: AB_1107636
InVivoMAb Rat IgG2b isotype control, anti-keyhole limpet hemocyanin	BioXCell	Cat# BE0090; RRID: AB_1107780
Alexa 594 conjugated Affinipure donkey anti-rabbit IgG	Jackson Immunoresearch	Cat# 711–585-152; RRID: AB_2340621
Alexa 488 conjugated Affinipure donkey anti-rat IgG	Jackson Immunoresearch	Cat# 712–545-150; RRID: AB_2340683

Bacterial and virus strains		

*Streptococcus pneumoniae* serotype 19F Strain EF3030	Smith et al.^63^	https://doi.org/10.1038/mi.2017.43
*Streptococcus pneumoniae* serotype 3 Strain ATCC 6303	ATCC	ATCC 6303

Chemicals, peptides, and recombinant proteins		

Albumin from chicken egg white (Ovalbumin)	Sigma Aldrich	Product# A5503–10G
Imject^™^ Alum Adjuvant	Thermo Scientific^™^	Catalog# 77161
Ketamine	Covetrus	N/A
Xylazine	Covetrus	SKU# 061035
Methacholine	Sigma Aldrich	Cat# A2251–25G
Tamoxifen	Sigma Aldrich	Product# T5648–1G
Dexamethasone	Sigma Aldrich	Product# D4902–25MG
16% Paraformaldehyde aqueous solution	Fisher Scientific	Cat# 50–980-487
Phorbol 12-myristate 13-acetate (PMA)	Sigma Aldrich	Product# P8139–1MG
Ionomycin	Sigma Aldrich	Product# I0634–1MG
Monensin	Biolegend	Cat# 420701
Brefeldin A	Biolegend	Cat# 420601
TNF- α recombinant protein	R&D Systems	Cat# 410-MT-025
IFN- γ recombinant protein	R&D Systems	Cat# 485-MI-100
IL-17A recombinant protein	R&D Systems	Cat# 421-ML-025
DAPI	Molecular Probes Life Technologies	Cat# R37606
FluorSave^™^ Reagent	Millipore Calbiochem	Cat# 345789
Corn Oil	Sigma Aldrich	Cat# C8267–500ML

Critical commercial assays		

Mouse LIX DuoSet ELISA	R&D Systems	Cat# DY443
Mouse IL-17 DuoSet ELISA	R&D Systems	Cat# DY421
Mouse IFN-γ DuoSet ELISA	R&D Systems	Cat# DY485
Mouse CXCL1/KC DuoSet ELISA	R&D Systems	Cat# DY453
Mouse CXCL2/MIP-2 DuoSet ELISA	R&D Systems	Cat# DY452
Mouse CXCL10/IP-10/CRG-2 DuoSet ELISA	R&D Systems	Cat# DY466
RNAeasy Micro Kit	Qiagen	Cat# 74004
TaqMan^™^ RNA-to-CT^™^ 1-Step Kit	Life Technologies	Cat# 4392938
eBioscience Foxp3/Transcription Factor Staining Buffer Set	Invitrogen	Cat# 00–5523-00
eBioscience Intracellular Fixation & Permeabilization Buffer Set	Invitrogen	Cat# 88–8824-00

Experimental models: Cell lines		

Mouse lung epithelial Cells (MLE12)	ATCC	ATCC CRL-2110

Experimental models: Organisms/strains		

C57BL/6J	The Jackson Laboratory	Stock# 000664
B6.Cg-*Il17a/il17f^tm1.1Impr^ Thy1^a^*/J	The Jackson Laboratory	Stock# 034140
B6.129S7-*Ifng^tm1Ts^*/J	The Jackson Laboratory	Stock# 002287
Nkx2-1^tm1.1(cre/ERT2)Zjh^/J	The Jackson Laboratory	Stock# 014552
B6.129X1-H2-Ab1^tm1Koni^/J	The Jackson Laboratory	Stock# 013181

Oligonucleotides		

Mouse Cxcl5 (Mm00436451_g1)	Thermofisher Scientific	Cat# 4331182
Mouse Cxcl1 (Mm04207460_m1)	Thermofisher Scientific	Cat# 4331182
Mouse Cxcl2 (Mm00436450_m1)	Thermofisher Scientific	Cat# 4331182
Mouse Cxcl10 (Mm00445235_m1)	Thermofisher Scientific	Cat# 4331182
Mouse Muc5ac (Mm01276718_m1)	Thermofisher Scientific	Cat# 4331182
Eukaryotic 18S rRNA Endogenous Control	Thermofisher Scientific	Cat# 4319413E

Software and algorithms		

FlowJo software	BD Biosciences	N/A
SpectraFlo	Cytek	N/A
Omiq.ai cloud computation platform (Omiq)	Shenoy et al.^69^	Methods;https://doi.org/10.1038/s41467-021-26045-w
Phenograph algorithm	Levine et al.^67^	https://doi.org/10.1016/j.cell.2015.05.047
opt-SNE space	Belkina et al.^66^	https://doi.org/10.1038/s41467-019-13055-y
Prism	Graphpad	N/A

Other		

Legacy FlexiVent	SCIREQ	N/A

## Highlights

- Recurrent OVA inhalation experience predisposes mice to rapid allergic airway neutrophilia
- Neutrophil-prone allergic lungs harbor CD4^+^ T_RM_ cells, including RORγt^negative/low^ T_H_17 cells
- Muc5ac^high^ secretory cells instruct CD4^+^ T_RM_ fates and neutrophilia via MHC class II and CXCL5
- Prophylactic or therapeutic delivery of IFN-γ curbs allergic airway neutrophilia
