# Supplementary material for: Lung CD4+ resident memory T cells use airway secretory cells to stimulate and regulate onset of allergic airway neutrophilic disease
Source: Cell Rep. Author manuscript; Available in PMC 2025 Apr 21. (PMC12011213; doi:10.1016/j.celrep.2025.115294)
Supplement: 1 [file NIHMS2069107-supplement-1.pdf]

Cell Reports, Volume *44*

Supplemental information

Lung CD4$^+$ resident memory T cells use airway secretory cells to stimulate and regulate onset of allergic airway neutrophilic disease

Vijay Raaj Ravi, Filiz T. Korkmaz, Carolina Lyon De Ana, Lu Lu, Feng-Zhi Shao, Christine V. Odom, Kimberly A. Barker, Aditya Ramanujan, Emma N. Niszczak, Wesley N. Goltry, Ian M.C. Martin, Catherine T. Ha, Lee J. Quinton, Matthew R. Jones, Alan Fine, Joshua D. Welch, Felicia Chen, Anna C. Belkina, Joseph P. Mizgerd, and Anukul T. Shenoy

Supplemental Information

Lung $CD4^{+}$ resident memory T cells use airway secretory cells to stimulate and regulate onset of allergic airways neutrophilic disease.

Vijay Raaj Ravi, Filiz T. Korkmaz, Carolina Lyon De Ana, Lu Lu, Feng-Zhi Shao, Christine V. Odom, Kimberly A. Barker, Aditya Ramanujan, Emma N. Niszczak, Wesley N. Goltry, Ian M.C. Martin, Catherine T. Ha, Lee J. Quinton, Matthew R. Jones, Alan Fine, Joshua D. Welch, Felicia Chen, Anna C. Belkina, Joseph P. Mizgerd, and Anukul T. Shenoy

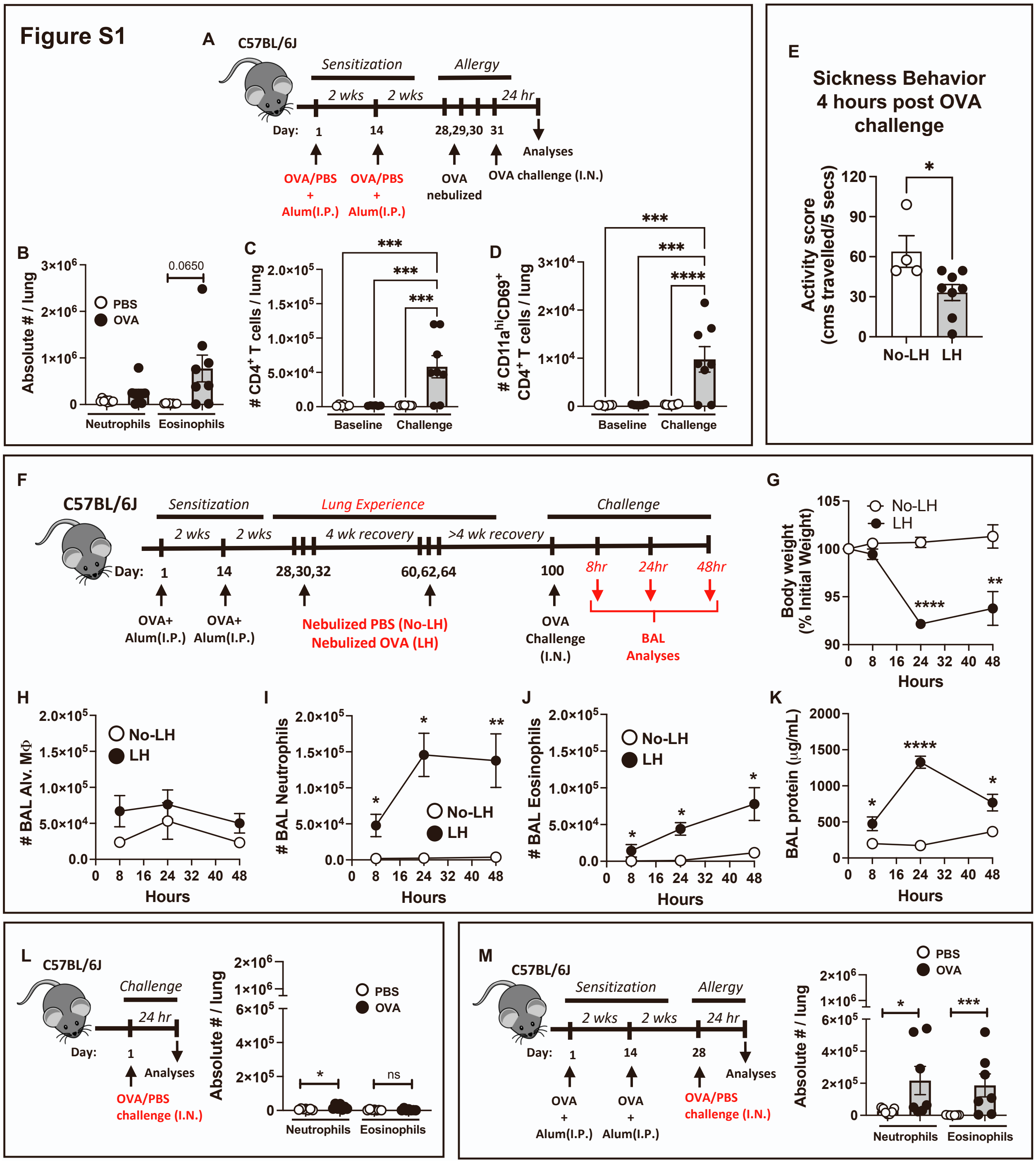

Figure S1: Comparisons of mouse models of OVA-induced eosinophilic and neutrophilic asthma. A) Schematic of experimental timeline used. **B)** Numbers of lung (ivCD45.2$^-$) neutrophils and eosinophils 24 hours post final OVA challenge in OVA or PBS sensitized C57BL6/J mice. Mann-Whitney test. **C-D)** Numbers of **C)** lung (ivCD45.2$^-$) CD4$^+$ T cells and **D)** lung (ivCD45.2$^-$) CD11a^{hi}CD69$^+$ CD4$^+$ T cells at baseline and 24 hours post OVA challenge. One-way ANOVA with Fisher's LSD Test. **E)** Activity score of LH and No-LH mice measured as distance travelled in cms within 5 seconds when

assessed 4 hours post OVA challenge. Mann-Whitney test. **F)** Schematic of experimental timeline used. **G)** Body weight of of LH and No-LH mice at designated timepoint. Mann-Whitney test comparing LH to No-LH at each timepoint. Total number of **H)** alveolar macrophages, **I)** neutrophils, and **J)** eosinophils in bronchoalveolar lavages (BAL) from LH and NoLH mice at designated timepoints after OVA challenge. Mann-Whitney test comparing LH and No-LH mice at each timepoint. **Note**: The Y axes in **Figs S1H-J** are adjusted to a similar scale to facilitate direct comparisons of airway inflammatory profiles at each timepoints. **K)** Lung damage expressed as BAL protein content. Unpaired t test. **L)** Schematic of experimental timeline used and numbers of lung (ivCD45.2$^-$) neutrophils and eosinophils 24 hours post PBS or OVA challenge in naïve C57BL6/J mice. Mann-Whitney test. **M)** Schematic of experimental timeline used and numbers of lung (ivCD45.2$^-$) neutrophils and eosinophils 24 hours post PBS or OVA challenge in OVA sensitized C57BL6/J mice. Mann-Whitney test. *p* value: *≤ 0.05, **≤ 0.01. All data have n≥3 mice, 2 experiments, mean ± SEM. Note: The Y axes in **Fig S1L** and **Fig S1M** are adjusted to be similar with **Fig 1H** to facilitate direct comparisons.

Figure S2

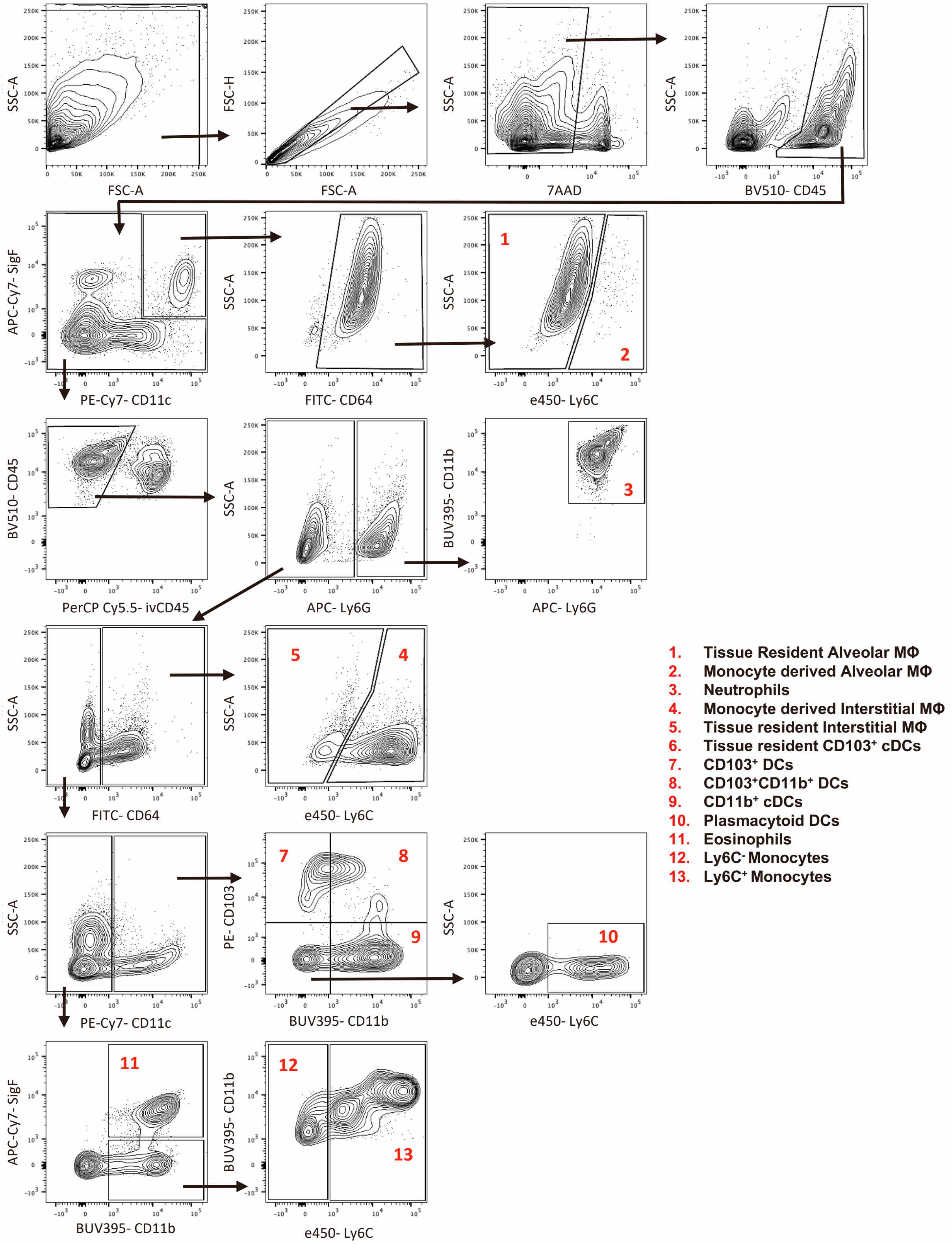

Figure S2: Gating strategy for comprehensive profiling of myeloid cells.

Figure S3

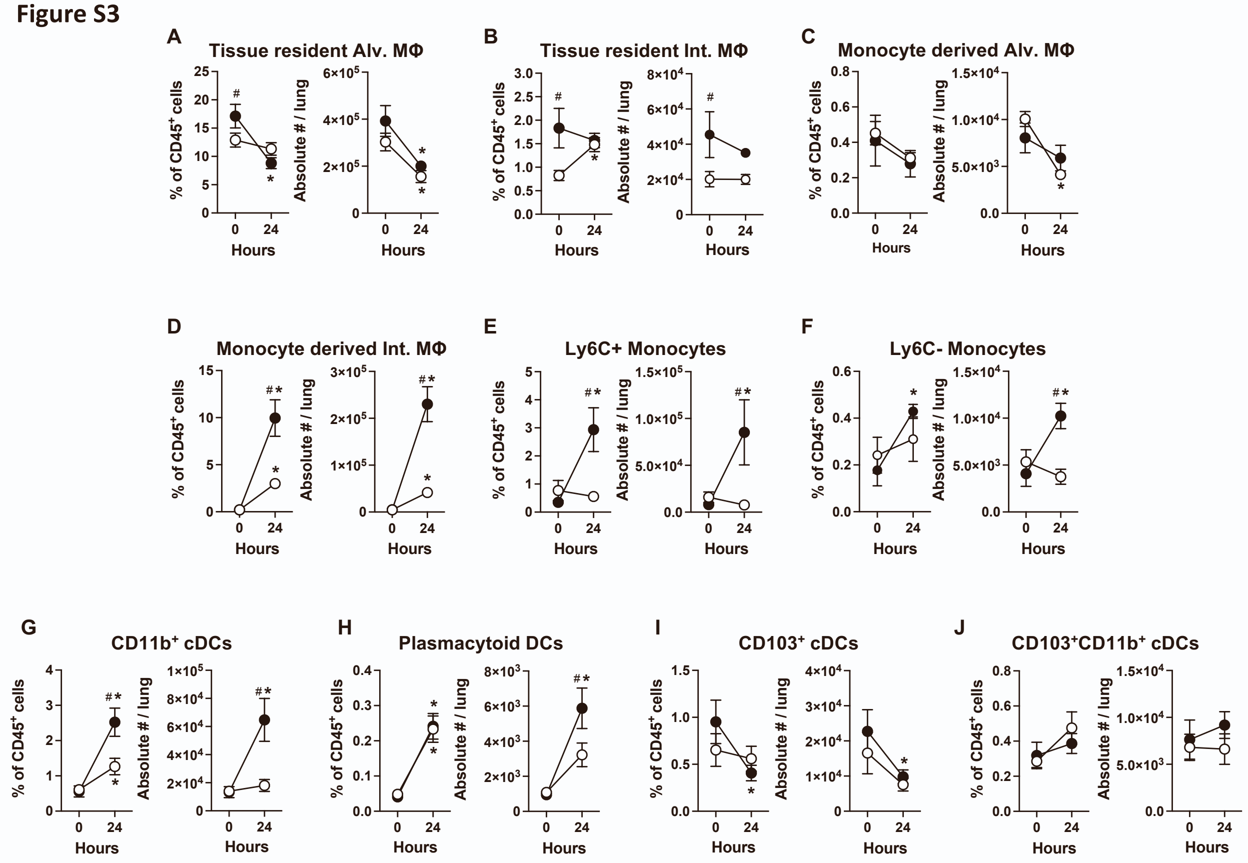

Figure S3: LH mice display extensive remodeling of lung myeloid landscape. Numbers of distinct lung (ivCD45.2⁻) myeloid cells at baseline and 24 hours post OVA challenge in no-LH (white) and LH mice (black), Two-way ANOVA with Fisher's LSD Test. *p* value: *≤ 0.05 for comparisons within same group across time-points and $^{\#}$≤ 0.05 for comparisons between groups at same time-point. All data have n≥5 mice, 2 experiments, mean ± SEM

Figure S4

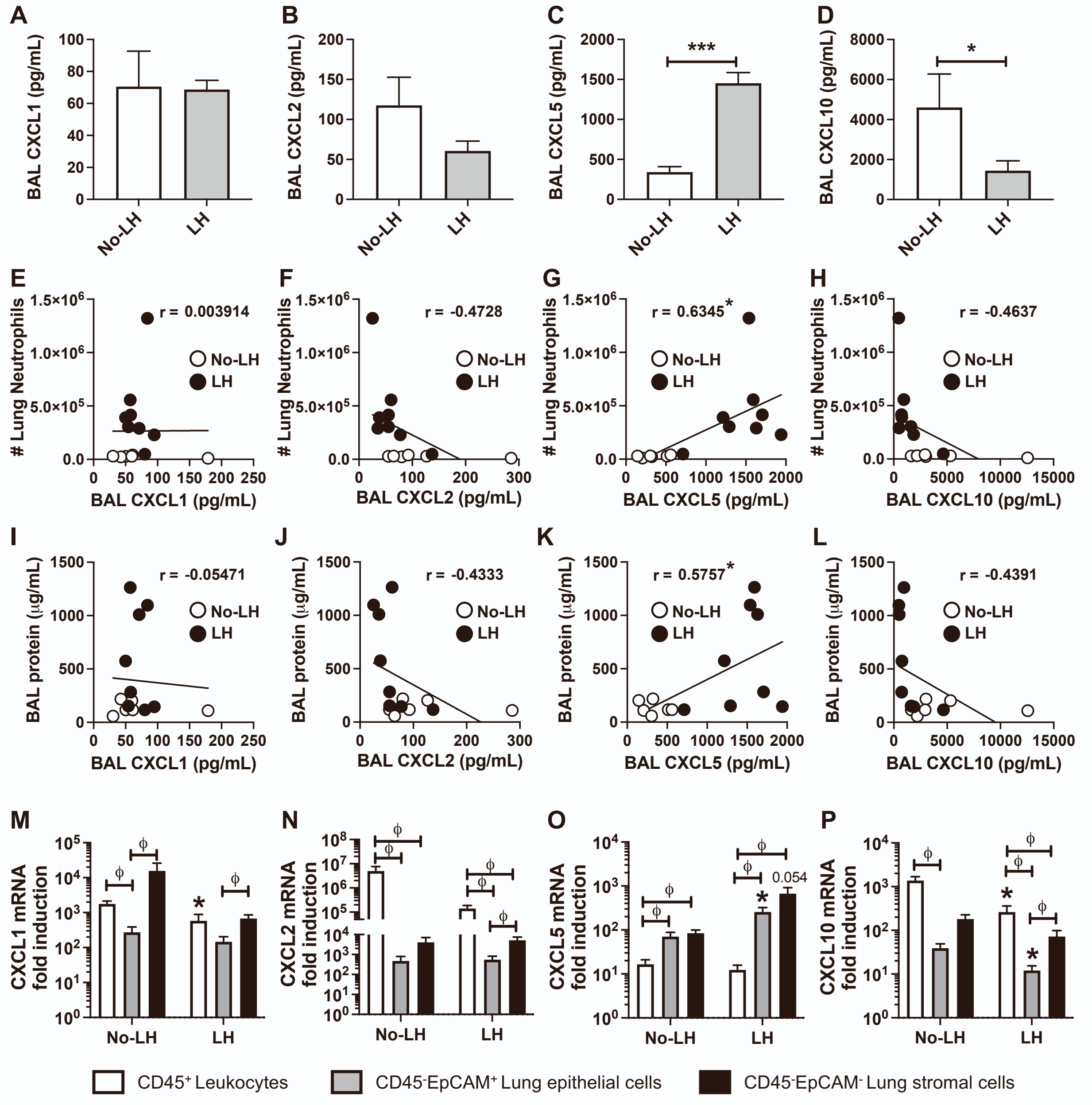

Figure S4: CXCL5 produced by lung epithelial and stromal cells correlates with severity of rapid allergic airways neutrophilia. A) CXCL1, **B)** CXCL2, **C)** CXCL5 and **D)** CXCL10 levels in BALs of mice 24 hours post OVA challenge. *p* value: ***≤ 0.001 by Mann-Whitney test. **E-H)** Scatterplots correlating lung neutrophil numbers with **E)** BAL CXCL1, **F)** BAL CXCL2, **G)** BAL CXCL5 and **H)** BAL CXCL10 levels in mice 24 hours post OVA challenge. Spearman's correlation coefficient (r) and statistical significance denoted. *p* value: *≤ 0.05. **I-L)** Scatterplots correlating lung damage (BAL protein) with **I)** BAL CXCL1, **J)** BAL CXCL2, **K)** BAL CXCL5 and **L)** BAL CXCL10 levels in mice 24 hours post OVA challenge. Spearman's correlation coefficient (r) and statistical significance

denoted. *p* value: *≤ 0.05. **M-P)** mRNA levels of **M)** CXCL1, **N)** CXCL2, **O)** CXCL5 and **P)** CXCL10 transcripts in FACS-sorted $CD45^+$ leukocytes (*white*), $CD45^-EpCAM^+$ lung epithelial cells (*gray*), $CD45^-EpCAM^+$ structural cells (*black*) from mice 24 hours post OVA challenge expressed as fold change induction over naïve whole lungs. *p* value: $^{\Phi}$≤ 0.05 by Kruskal-Wallis test between cell types but same treatment group, and *≤ 0.05 by Unpaired t test within a cell type but between treatment groups. All data have n≥5 mice, 2 experiments, mean ± SEM.

Figure S5

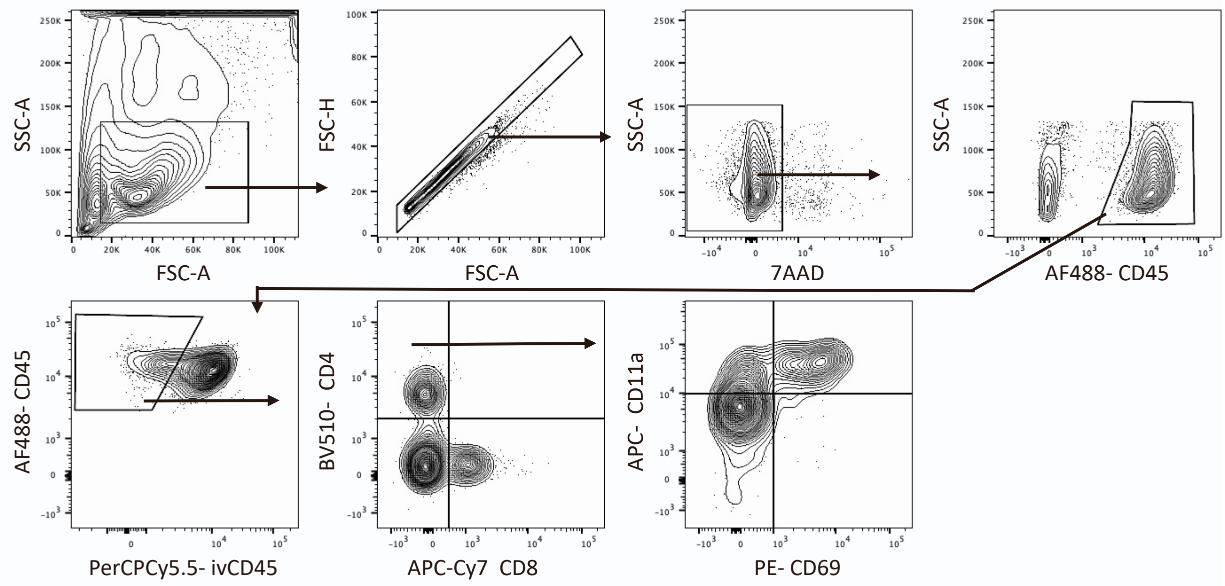

Figure S5: Gating strategy for identification of lung-resident CD4⁺ T_{RM} cells.

Figure S6

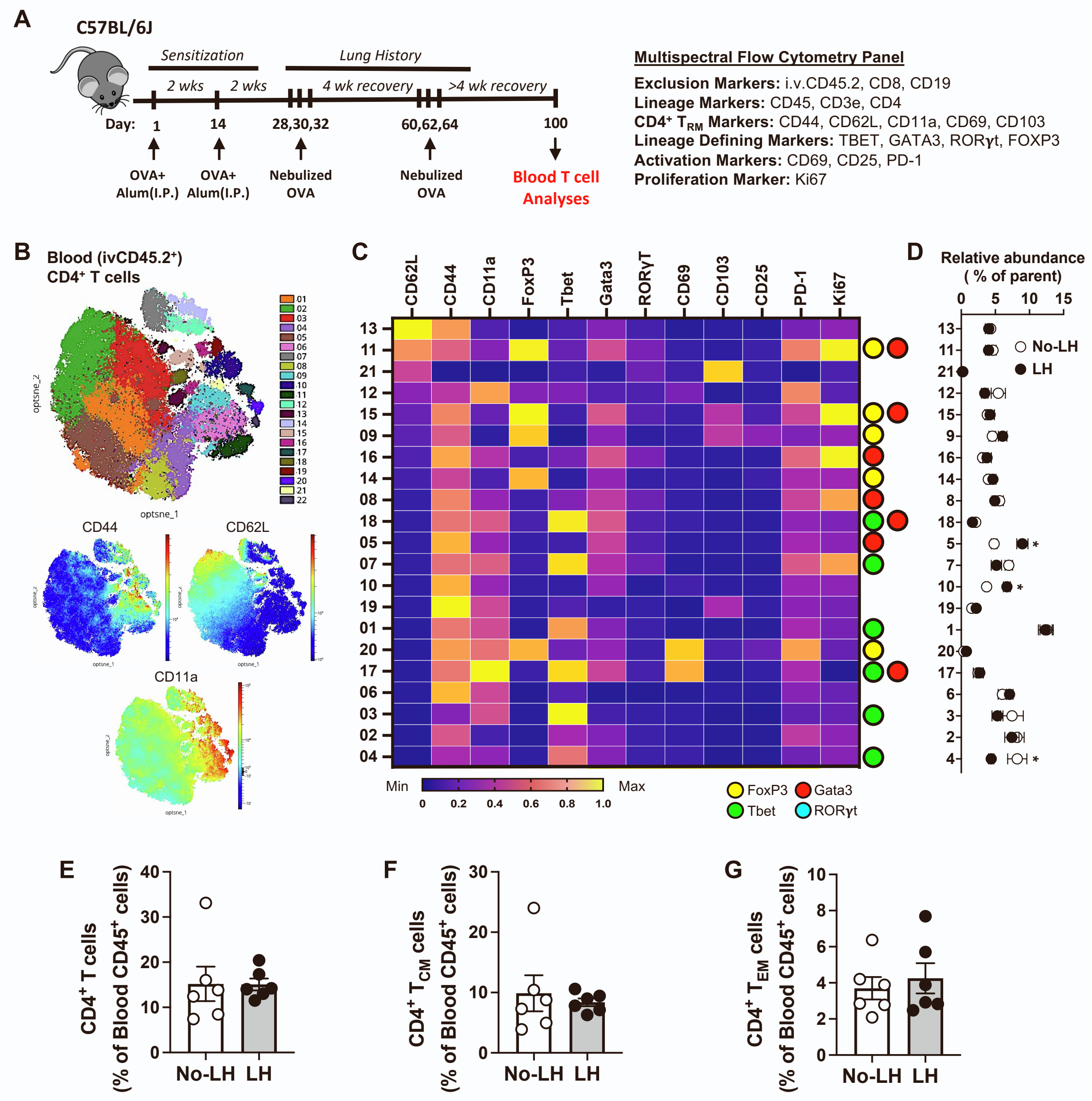

Figure S6: LH mice lack RORγt$^+$ T$_H$17 cells within circulation and share a comparable blood CD4$^+$ T cell pool with No-LH mice. A) Schematic of experimental timeline and antibody panel used. **B)** Phenograph clustering overlaid on opt-SNE projection depicting blood (i.v.CD45.2$^+$) CD4$^+$ T cells concatenated from n=8 LH mouse lungs across 2 experiments on day 100. opt-SNE projection with heatmap visualization depicting CD44, CD62L and CD11a expression levels are shown. **C)** Heat map depicting normalized expression levels of distinct molecules on blood (ivCD45.2$^+$) CD4$^+$ T cell clusters. Lineage determining transcription factor (LDTF) status of each cluster is depicted on the *right*. **D)** Relative abundances of each blood (ivCD45.2$^+$) CD4$^+$ T cell cluster in LH mice (white) and No-LH mice(black). Two Way ANOVA with two stage step-

up method of Benjamini, Krieger, and Yekutieli to correct for multiple comparisons. FDR *p* value: *≤0.05. Relative abundances of blood (ivCD45.2^{+}) **E)** CD4^{+} T cells, **F)** CD62L^{+}CD44^{+} CD4^{+} T_{CM} cells and **G)** CD62L^{-}CD44^{+} CD4^{+} T_{EM} cells expressed as percentage fraction of blood (ivCD45.2^{+}) CD45^{+} cells in LH mice (white) and No-LH mice (black). All data have n≥5 mice, 2 experiments, mean ± SEM.

Figure S7

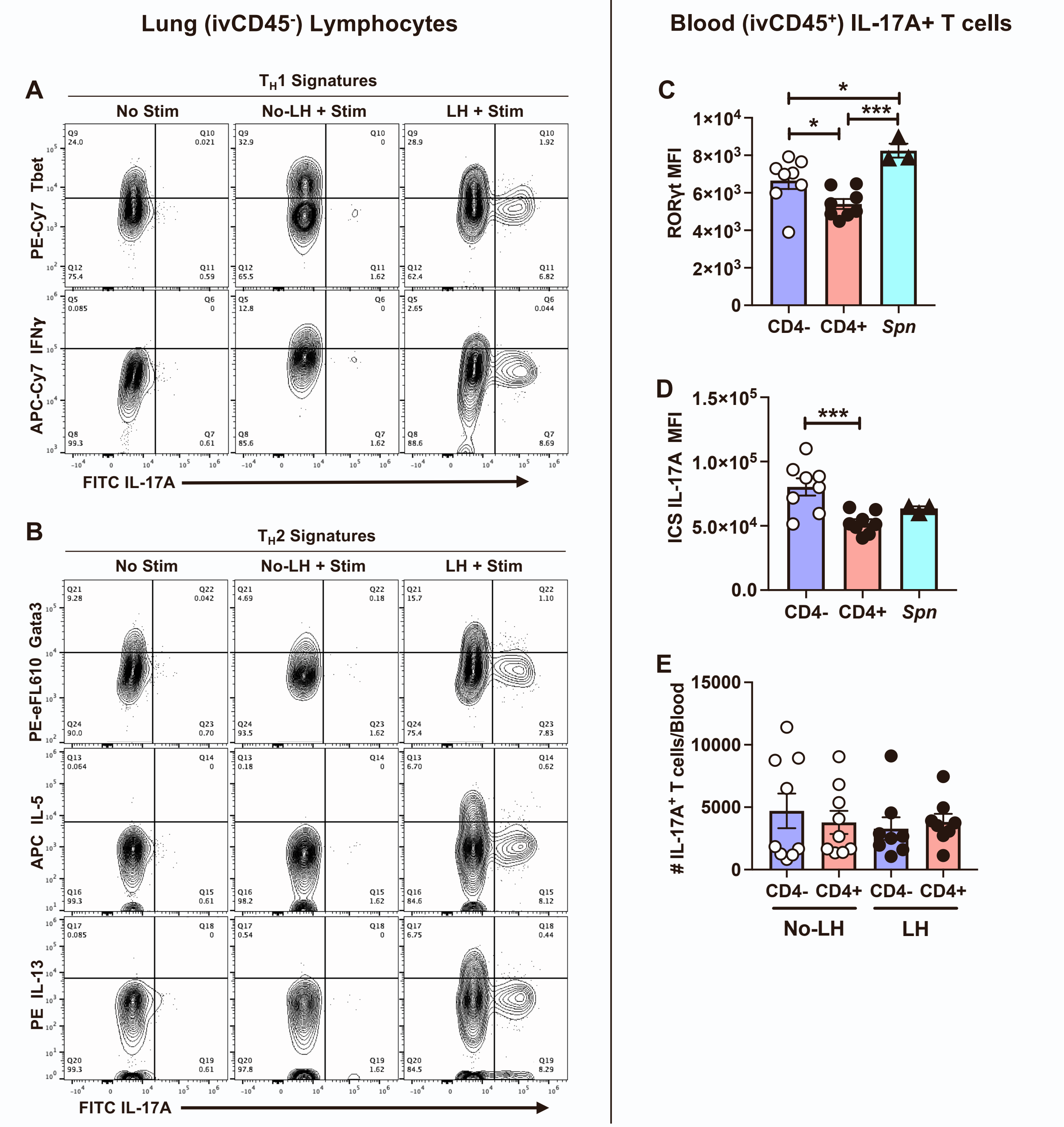

Figure S7: RORγt$^{negative/low}$ T$_H$17 T$_{RM}$ cells are distinct from other traditional helper cell subsets and not enriched in blood. Representative contour plots depicting **A)** Tbet and IFN-γ, and **B)** Gata3, IL-5 and IL-13 expression patterns in RORγt$^{negative/low}$ IL-17A$^+$ producing lung (ivCD45.2$^-$) CD4$^+$ T cells in LH mice. Expression levels of **C)** RORγt and **D)** IL-17A in IL-17A$^+$ blood (ivCD45.2$^+$) CD4$^-$ and CD4$^+$ T cells in LH mice. One-way ANOVA with Fisher's LSD Test. Expression patterns of *S. pneumoniae* induced (ivCD45.2$^+$) CD4$^+$ T cells are included as positive control for T$_H$17 signatures. **J)** Absolute

numbers of IL-17A$^+$ blood (ivCD45.2$^-$) CD4$^-$ and CD4$^+$ T cells in No-LH and LH mice. One-way ANOVA with Fisher's LSD Test. All data have n≥3 mice, 2 experiments, mean ± SEM. *p* value: *≤0.05, **≤ 0.01, ***≤ 0.001.

Figure S8

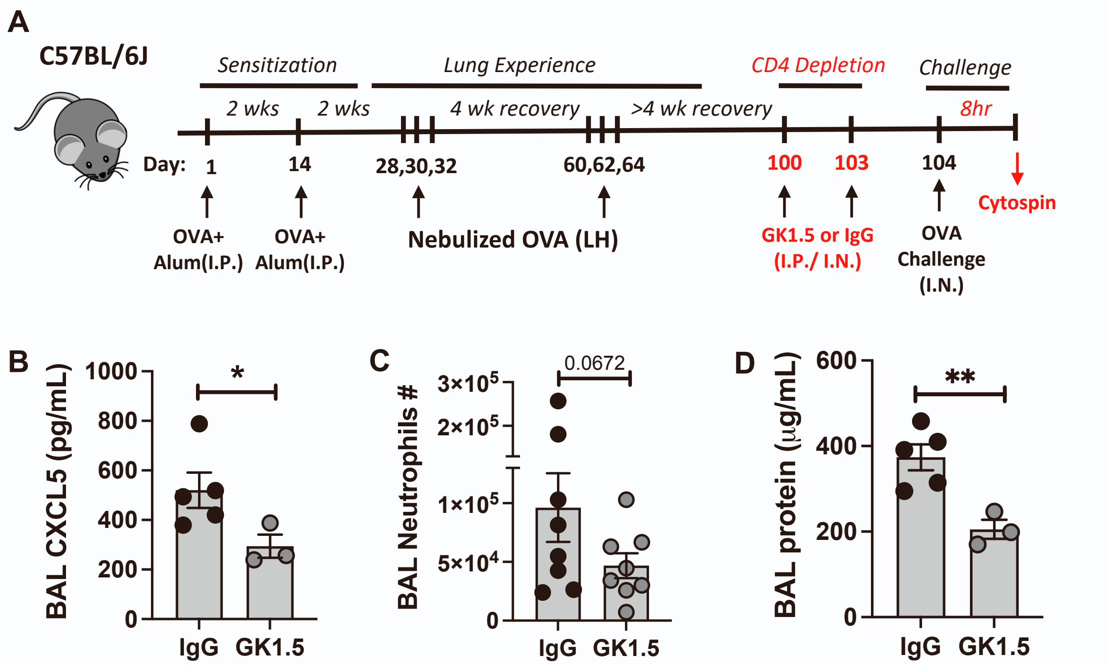

Figure S8: Depletion of CD4⁺ T$_{RM}$ cells preempts allergic airway neutrophilia. A) Schematic of experimental timeline used. **B)** CXCL5 levels, **C)** neutrophils numbers, and **D)** edema in BAL of GK1.5 (or IgG) treated LH mice 8-hours post OVA challenge. Data except **S8B** and **S8D** has n≥5 mice, 2 experiments, mean ± SEM, One-tailed Unpaired t-test. *p* value: *≤ 0.05, **≤ 0.01.

Figure S9

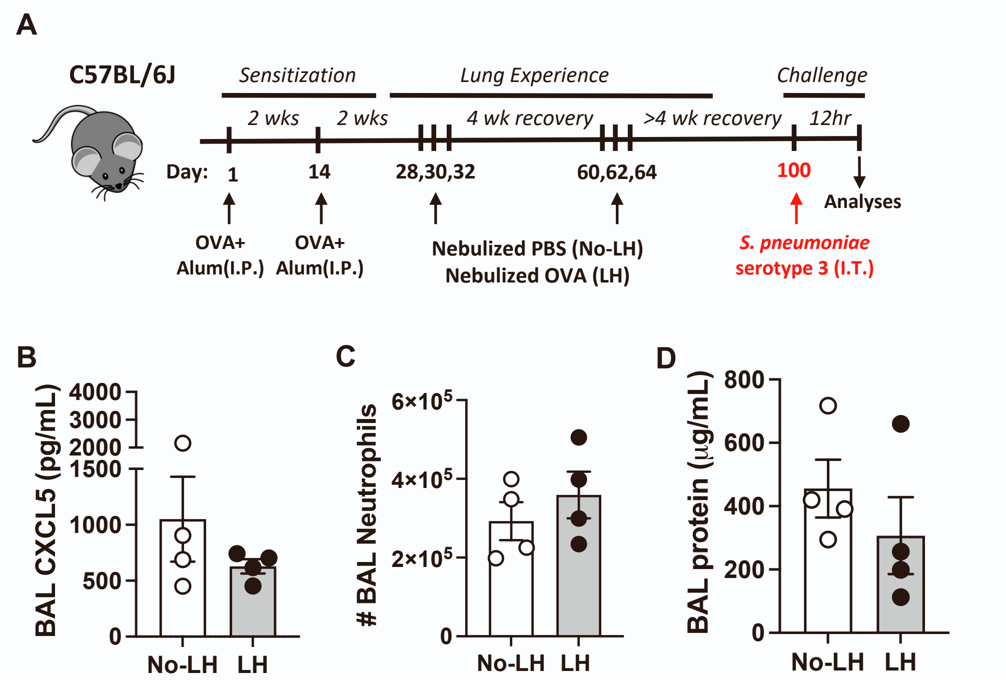

Figure S9: Allergic airway neutrophilia in LH mice is antigen specific and independent of trained immunity. A) Schematic of experimental timeline used. **B)** CXCL5 levels, **C)** neutrophils numbers, and **d)** edema in BAL of No-LH and LH mice 12-hours post *Streptococcus pneumoniae* serotype 3 challenge. *S. pneumoniae* was used as a model antigen irrelevant to the allergen to test role of trained immunity in the neutrophilic response. All data n=4 mice, 2 experiments, mean ± SEM.

Figure S10

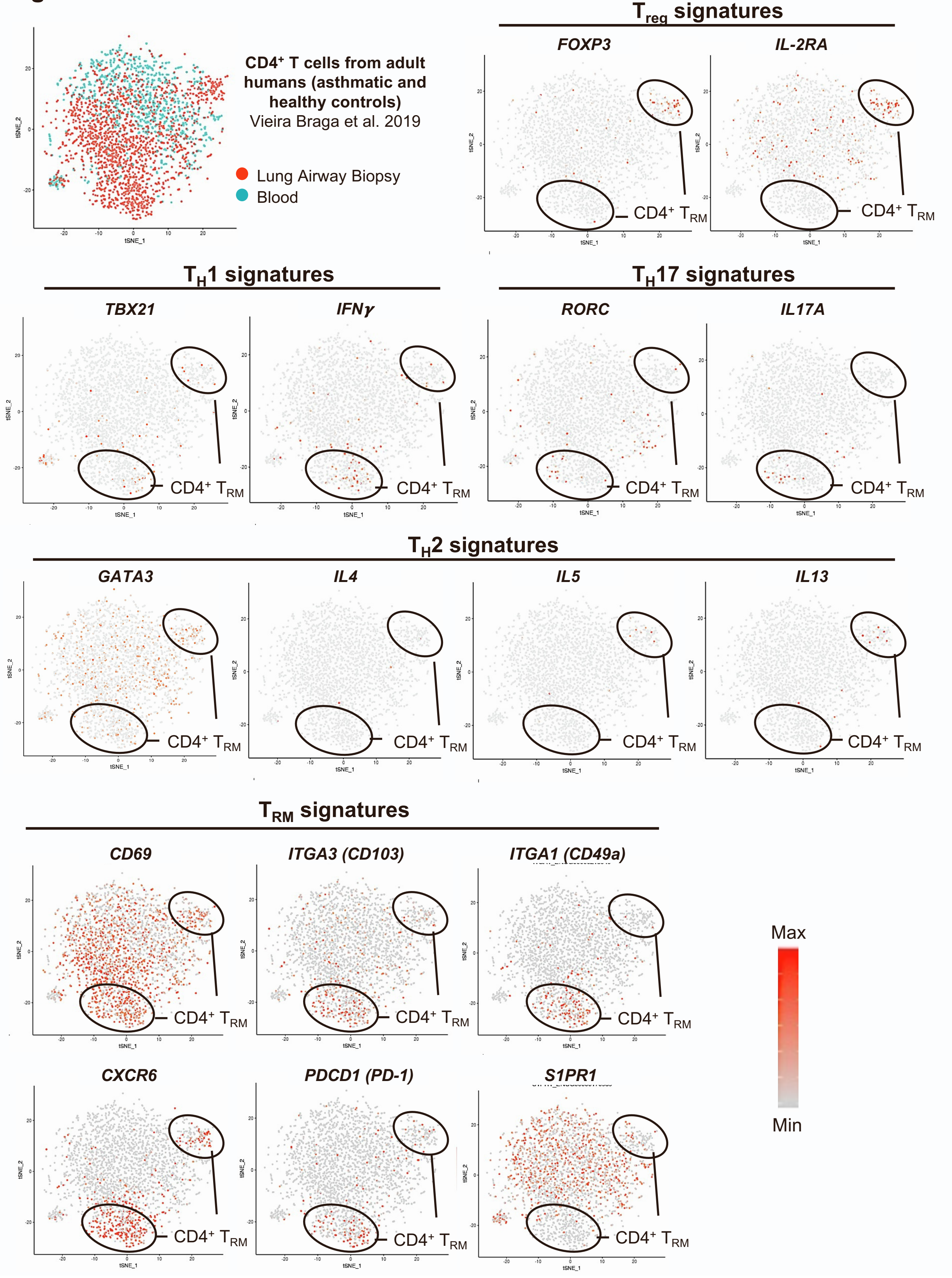

CD4+ T cells from adult humans (asthmatic and healthy controls)
Vieira Braga et al. 2019
Lung Airway Biopsy
Blood
Treg signatures
FOXP3
IL-2RA
TH1 signatures
TBX21
IFNγ
TH17 signatures
RORC
IL17A
TH2 signatures
GATA3
IL4
IL5
IL13
TRM signatures
CD69
ITGA3 (CD103)
ITGA1 (CD49a)
CXCR6
PDCD1 (PD-1)
S1PR1
CD4+ TRM
tSNE_1
tSNE_2
Max
Min

Figure S10: Airways of adult asthmatic and healthy human lungs harbor diverse clusters of $CD4^+$ T_{RM} cells including RORγt$^{negative/low}$ T_H17 T_{RM} cells. t-SNE projection of scRNA-Seq data depicting expression patterns for diverse markers of interest as expressed by $CD4^+$ T cell isolated from airway wall biopsies and peripheral blood of adult asthmatic and healthy humans and as displayed on the interactive web portal: https://asthma.cellgeni.sanger.ac.uk/ [1].

Figure S11

T cells from adult human airways (asthmatic and healthy controls) Alladina et al. Sci. Immunol. 2023

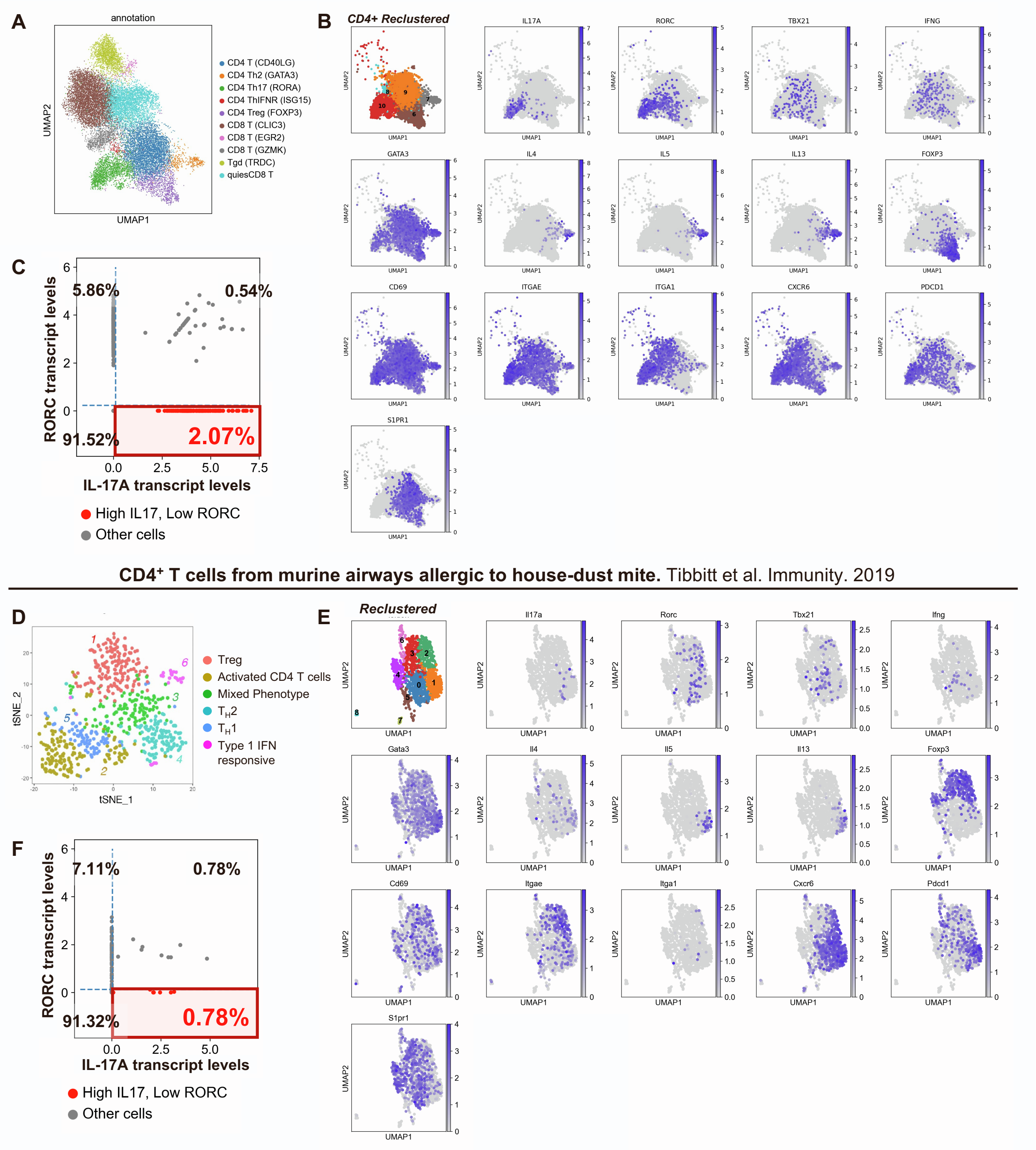

Figures S11: Airways of adult asthmatic and healthy humans (but not mice with allergic airways eosinophilia) are enriched for RORγt$^{negative/low}$ T$_H$17 T$_{RM}$ cells. A) UMAP projection of scRNA-Seq data- and **B)** expression patterns for diverse markers of interest- as expressed by CD4$^+$ T cell isolated from airway brushings of adult asthmatic 24 hours post segmental allergen challenge. **C)** Dot plot depiction of scRNA-Seq data showing frequencies of RORγt^{negative} T$_H$17 T$_{RM}$ cells in human airway brushings 24 hours

post segmental allergen challenge. **Fig S11A-C** is obtained by reanalyses of published dataset[2]. **D)** UMAP projection of scRNA-Seq data- and **E)** expression patterns for diverse markers of interest- as expressed by $CD4^+$ T cell isolated from airways (BAL) of mice exposed to HDM (house dust mites) induced allergic airways eoisnophilia. **F)** Dot plot depiction of scRNA-Seq data showing frequencies of $ROR\gamma t^{negative}$ T_H17 T_{RM} cells in mice with HDM-induced allergic airways eosinophilia. **Fig S11D-F** is obtained by reanalyses of published dataset[3].

Figure S12

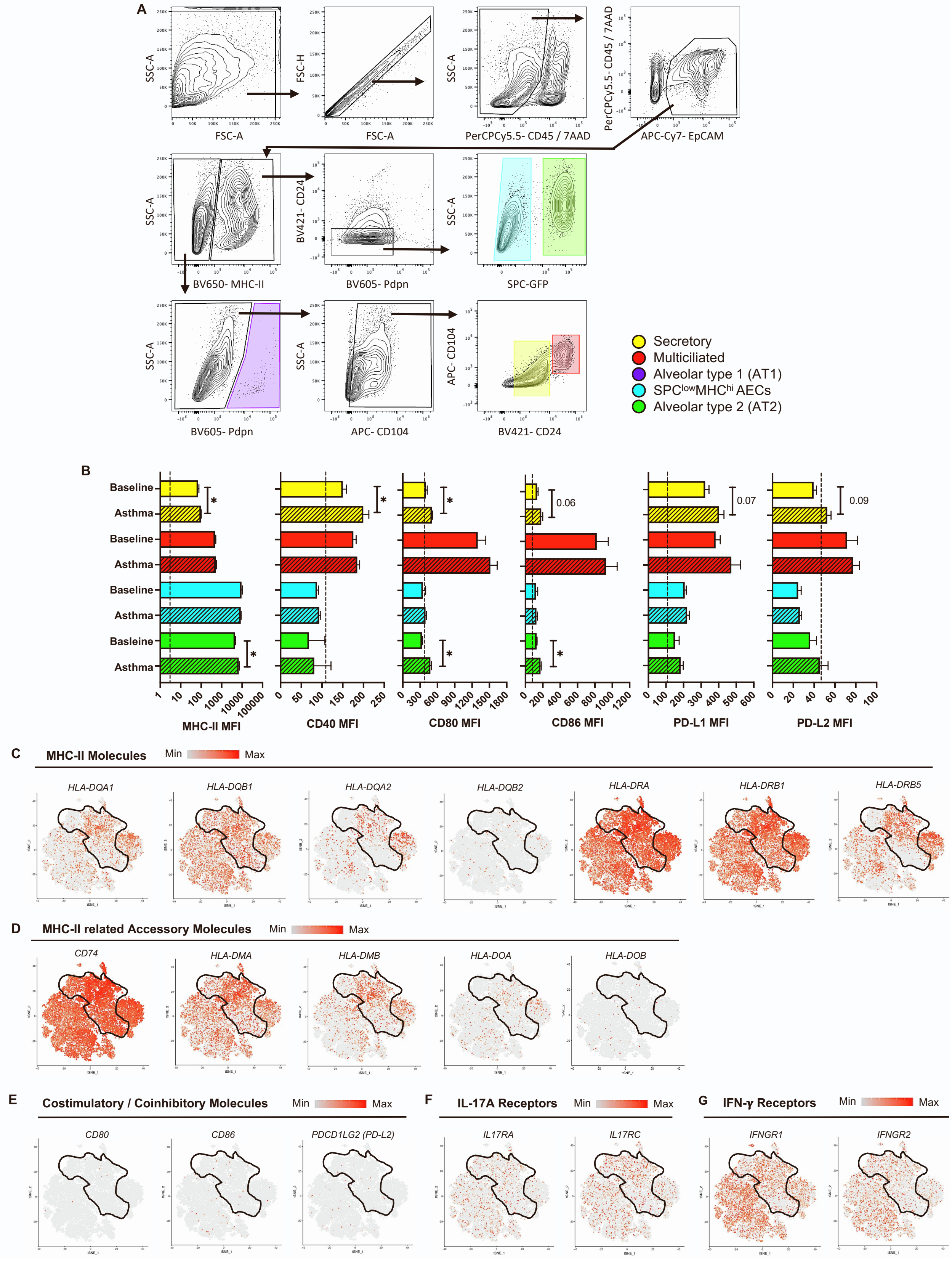

A
SSC-A
FSC-A
FSC-H
PerCPCy5.5- CD45 / 7AAD
APC-Cy7- EpCAM
BV650- MHC-II
BV421- CD24
BV605- Pdpn
SPC-GFP
APC- CD104
BV421- CD24
Secretory
Multiciliated
Alveolar type 1 (AT1)
SPClowMHChi AECs
Alveolar type 2 (AT2)
B
Baseline
Asthma
Baseline
Asthma
Baseline
Asthma
Basleine
Asthma
MHC-II MFI
CD40 MFI
CD80 MFI
CD86 MFI
PD-L1 MFI
PD-L2 MFI
0.06
0.07
0.09
C
MHC-II Molecules
Min
Max
HLA-DQA1
HLA-DQB1
HLA-DQA2
HLA-DQB2
HLA-DRA
HLA-DRB1
HLA-DRB5
D
MHC-II related Accessory Molecules
Min
Max
CD74
HLA-DMA
HLA-DMB
HLA-DOA
HLA-DOB
E
Costimulatory / Coinhibitory Molecules
Min
Max
CD80
CD86
PDCD1LG2 (PD-L2)
F
IL-17A Receptors
Min
Max
IL17RA
IL17RC
G
IFN-γ Receptors
Min
Max
IFNGR1
IFNGR2

Figure S12: Airway Muc5ac^{high} secretory cells can communicate with CD4$^+$ T$_{RM}$ cells in lungs with extensive inhaled allergen history. A) Gating strategy for identification of distinct lung epithelial cell (LEC) subsets in SPC-GFP LH mice. **B)** Surface expression levels of specified APC-related molecules on distinct epithelial cells from LH mice at baseline (clear bars) and 24 hours post OVA challenge (slashed bars). Unpaired t test. Dotted lines indicate signal level on FMO controls. Mann-Whitney test. *p* value: *≤ 0.05. All data have n=4 mice, 2 experiments, mean ± SEM. **C-G**) t-SNE projection of scRNA-Seq data depicting expression patterns for designated markers of interest by different subsets of epithelial cells identified in airways of adult asthmatics and healthy humans as displayed on the interactive web portal: https://asthma.cellgeni.sanger.ac.uk/ [1].

Figure S13

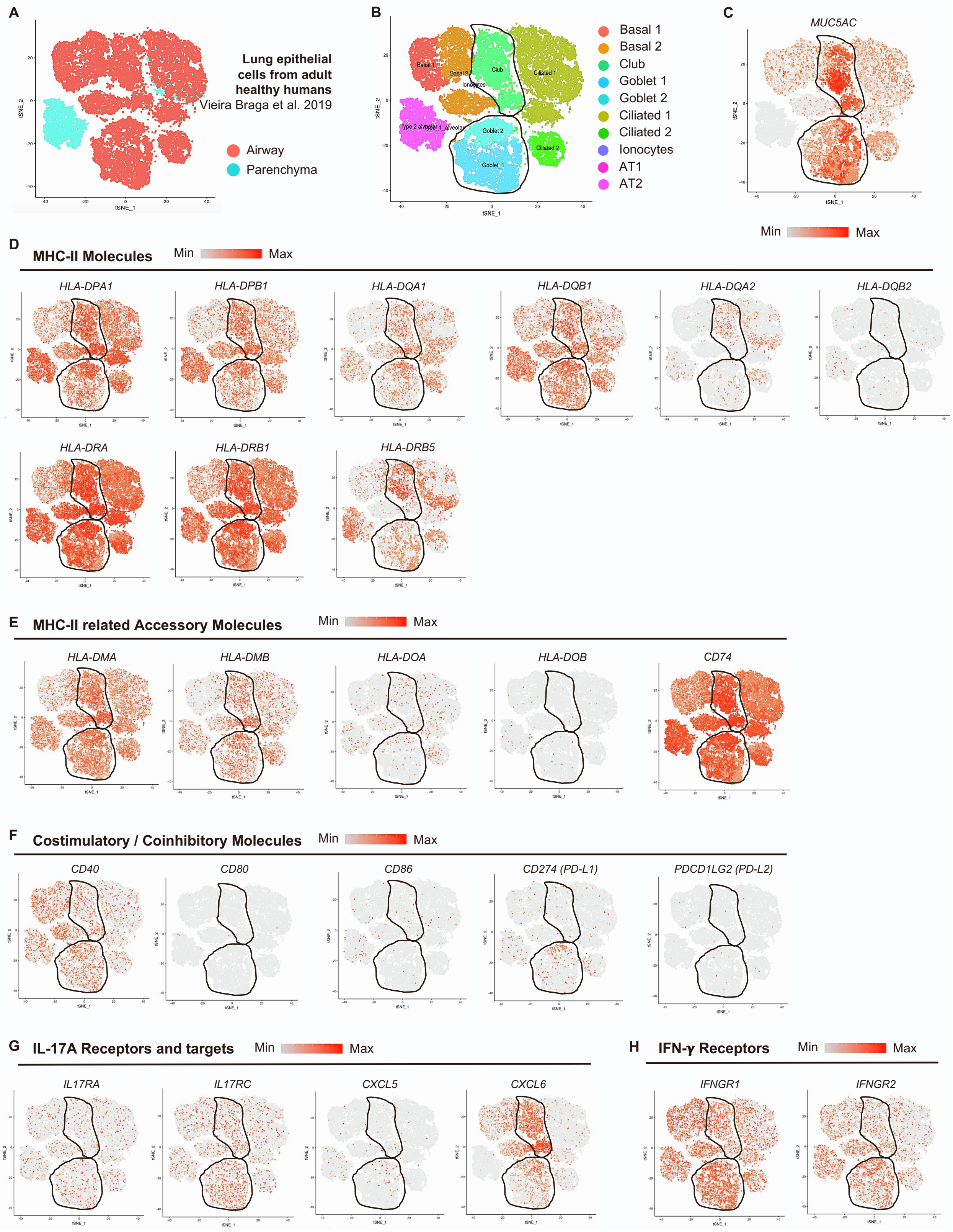
A
Lung epithelial cells from adult healthy humans
Vieira Braga et al. 2019
Airway
Parenchyma
B
Basal 1
Basal 2
Club
Goblet 1
Goblet 2
Ciliated 1
Ciliated 2
Ionocytes
AT1
AT2
C
MUC5AC
Min
Max
D
MHC-II Molecules
Min
Max
HLA-DPA1
HLA-DPB1
HLA-DQA1
HLA-DQB1
HLA-DQA2
HLA-DQB2
HLA-DRA
HLA-DRB1
HLA-DRB5
E
MHC-II related Accessory Molecules
Min
Max
HLA-DMA
HLA-DMB
HLA-DOA
HLA-DOB
CD74
F
Costimulatory / Coinhibitory Molecules
Min
Max
CD40
CD80
CD86
CD274 (PD-L1)
PDCD1LG2 (PD-L2)
G
IL-17A Receptors and targets
Min
Max
IL17RA
IL17RC
CXCL5
CXCL6
H
IFN-γ Receptors
Min
Max
IFNGR1
IFNGR2

Figure S13: Muc5ac^{high} airway secretory cells are high expressors of CD4^{+} T cell and neutrophil facing molecules. t-SNE projection of scRNA-Seq data depicting expression patterns for designated markers of interest by different subsets of epithelial cells identified in airways and lung parenchyma of adult humans as displayed on the interactive web portal: https://asthma.cellgeni.sanger.ac.uk/ [1].

Figure S14

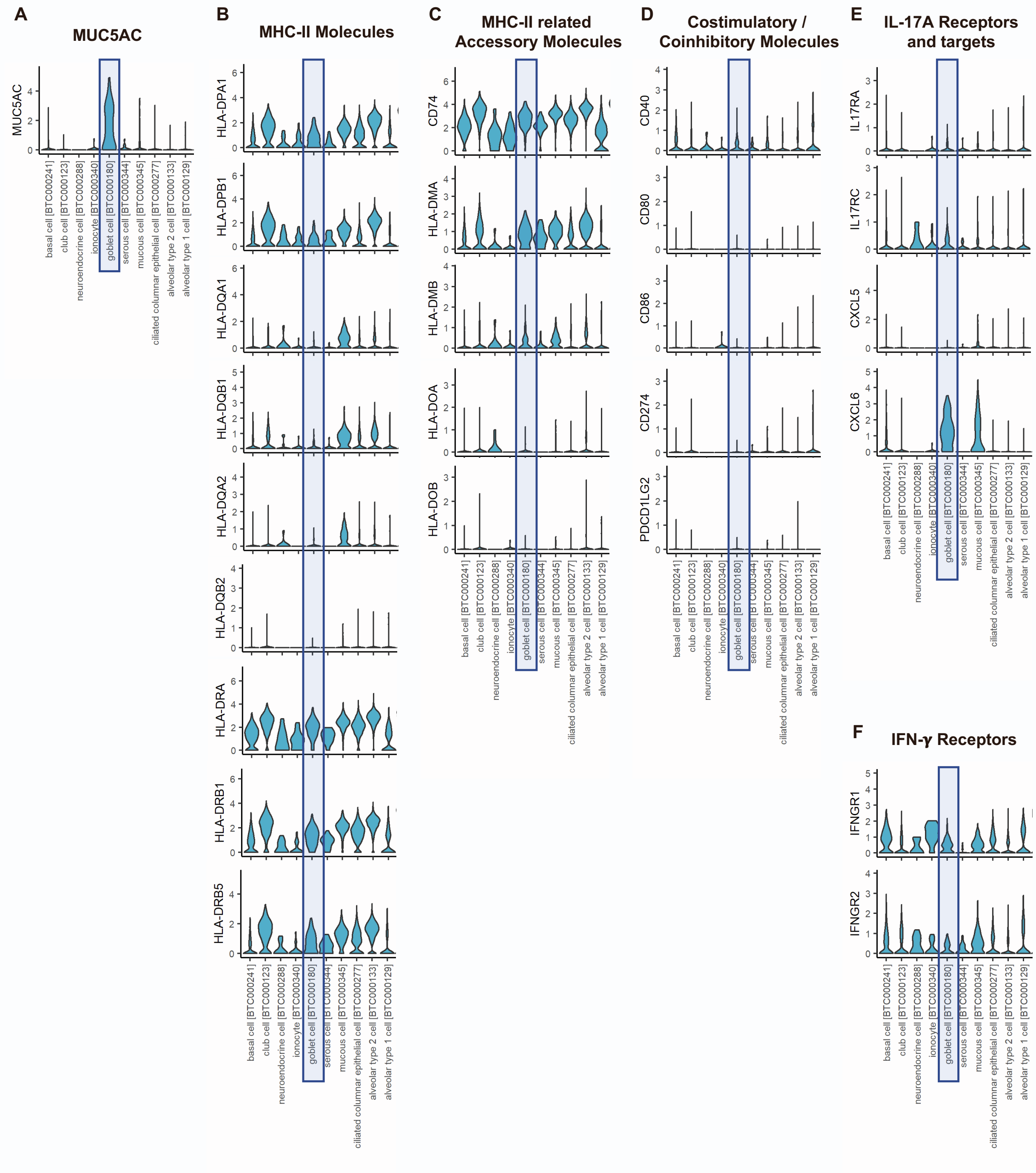

Figure S14: Muc5ac^{high} airway secretory cells may bridge CD4^{+} T$_{RM}$ cells and neutrophils in lungs with extensive inhalation histories. t-SNE projection of scRNA-Seq data depicting expression patterns for designated markers of interest by different subsets of epithelial cells identified in lungs of adult humans [4].

Figure S15

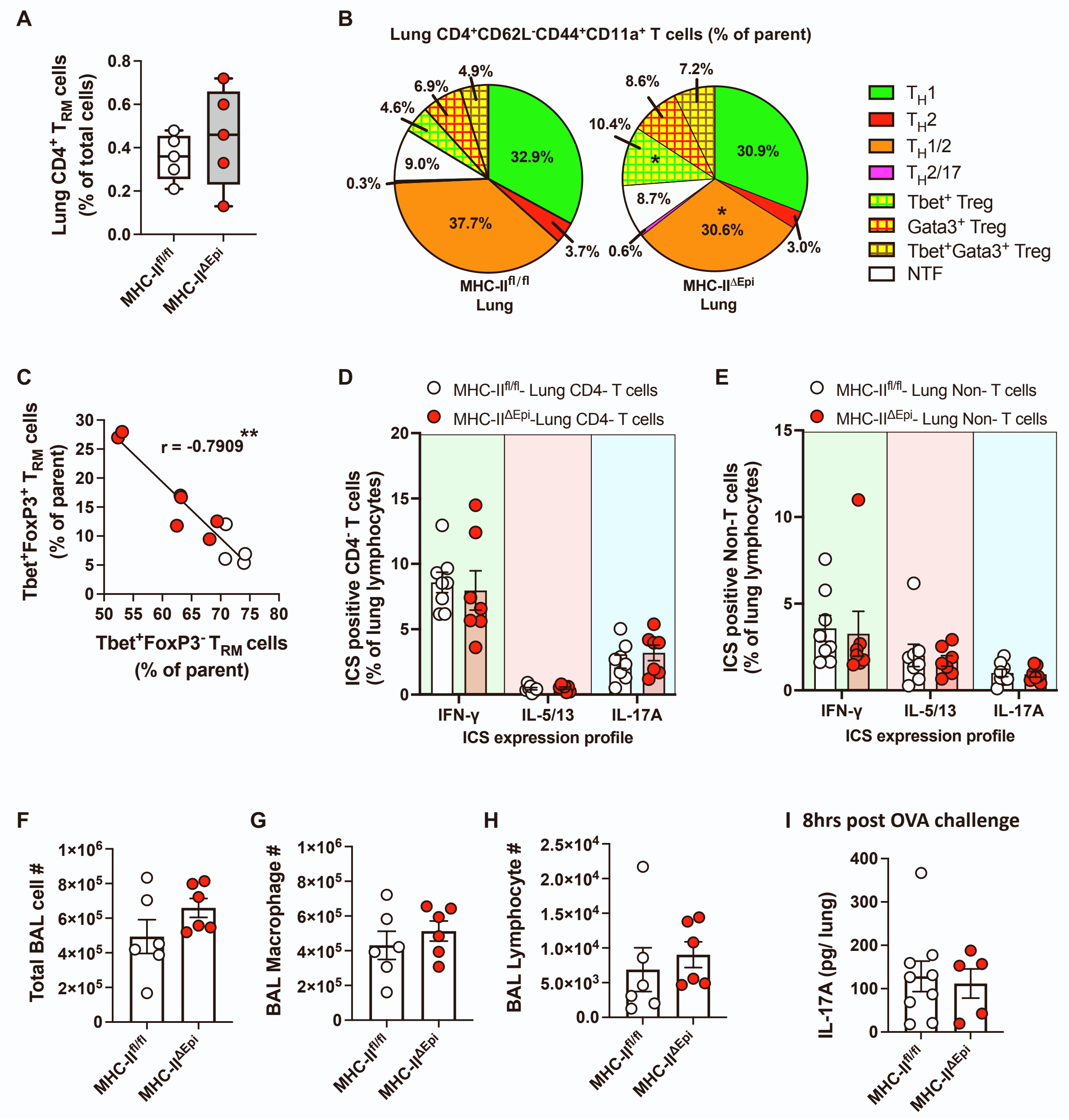

Figure S15: Characterization of LH mice lacking MHC-II specifically in lung epithelial cells. A) Abundance of lung CD4$^+$ T_{RM} cell numbers at baseline. **B)** Pie charts for mean frequencies of distinct memory T_H cell lineages within lungs of MHC-II$^{fl/fl}$ and MHC-II$^{\Delta Epi}$ LH mice at day 120, Two-way ANOVA with Fisher's LSD test. p-value: *≤ 0.05. **C)** Scatterplot correlating fraction all Tbet$^+$ FoxP3$^-$ CD4$^+$ T_{RM} cells with Tbet$^+$ FoxP3$^+$ T_{RM} cells. Spearman's correlation coefficient (r) and statistical significance denoted. Positivity for a tested LDTF was determined using cutoffs identified from clusters negative for that LDTF. **D, E)** Intracellular cytokine staining (ICS) profile of **D)** lung (i.v.CD45.2$^-$) CD4$^-$ T

cells and **E)** lung (i.v.CD45.2^{-}) CD3^{-} non-T cells isolated from lungs on day 120 and stimulated with PMA/Ionomycin *ex vivo*. Two-way ANOVA with Fisher's LSD Test. **F)** BAL cellularity-, **G)** BAL macrophage-, and **H)** BAL lymphocyte- numbers at 24 hours post OVA challenge. **I)** Levels of whole lung IL-17A in mice 8 hours post OVA challenge. All data have n≥4 mice, 2-3 experiments, mean ± SEM.

Figure S16

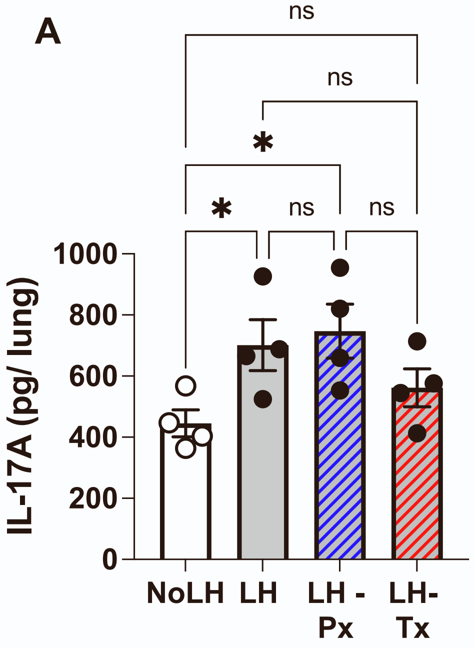

Figure S16: Exogenous supplementation of IFN-γ does not affects IL-17A levels in LH mice. A) Levels of whole lung IL-17A in mice 24 post OVA challenge with specified treatment modalities. Ordinary One-Way ANOVA with Fisher's LSD Test. All data have n=4 mice, 2 experiments, mean ± SEM. p-value: *≤ 0.05.

Figure S17

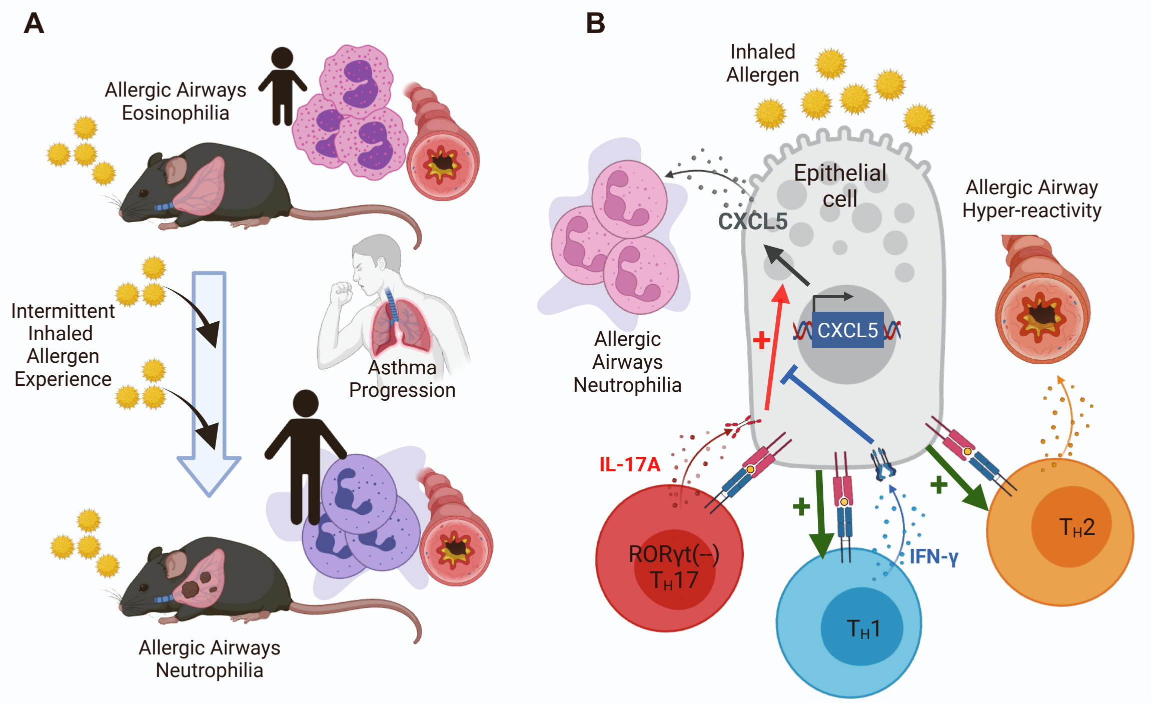

Figure S17: Proposed model. A) Intermittent exposures of sensitized mice to inhaled allergens over extended duration induces progression of eosinophilic allergic airways disease to enhanced neutrophilic allergic airways disease that recapitulates hallmark features of destructive neutrophilic asthma. **F)** Lungs of mice with age-appropriate inhaled allergen exposure history harbor diverse clusters of tissue-resident $CD4^{+}$ T_{RM} cells which includes T_H1, T_H2, and unconventional $ROR\gamma t^{neg/low}$ T_H17 T_{RM} cells. Antigenic reactivation of the latter leads to secretion of IL-17A, inducing expression of epithelial CXCL5 which elicits peribronchial neutrophilic infiltration in association with more severe disease. Antigen presentation by epithelial MHC-II bolsters T_H2 T_{RM} cells augmenting airway hyper-reactivity, and T_H1 T_{RM} cells; the latter via its effector cytokine IFN-γ curbs CXCL5 and airway neutrophilia driven by IL-17A. Thus, lung epithelial cells instruct $CD4^{+}$ T_{RM} cell fates and activities to control the type and extent of airway allergic responses.

REFERENCES

1. Vieira Braga, F.A., Kar, G., Berg, M., Carpaij, O.A., Polanski, K., Simon, L.M., Brouwer, S., Gomes, T., Hesse, L., Jiang, J., et al. (2019). A cellular census of human lungs identifies novel cell states in health and in asthma. Nat Med *25*, 1153-1163. 10.1038/s41591-019-0468-5.
2. Alladina, J., Smith, N.P., Kooistra, T., Slowikowski, K., Kernin, I.J., Deguine, J., Keen, H.L., Manakongtreecheep, K., Tantivit, J., Rahimi, R.A., et al. (2023). A human model of asthma exacerbation reveals transcriptional programs and cell circuits specific to allergic asthma. Sci Immunol *8*, eabq6352. 10.1126/sciimmunol.abq6352.
3. Tibbitt, C.A., Stark, J.M., Martens, L., Ma, J., Mold, J.E., Deswarte, K., Oliynyk, G., Feng, X., Lambrecht, B.N., De Bleser, P., et al. (2019). Single-Cell RNA Sequencing of the T Helper Cell Response to House Dust Mites Defines a Distinct Gene Expression Signature in Airway Th2 Cells. Immunity *51*, 169-184 e165. 10.1016/j.immuni.2019.05.014.
4. Travaglini, K.J., Nabhan, A.N., Penland, L., Sinha, R., Gillich, A., Sit, R.V., Chang, S., Conley, S.D., Mori, Y., Seita, J., et al. (2020). A molecular cell atlas of the human lung from single-cell RNA sequencing. Nature *587*, 619-625. 10.1038/s41586-020-2922-4.
